# Global Proteomic Response of *Caenorhabditis elegans* Against PemK_Sa_ Toxin

**DOI:** 10.3389/fcimb.2019.00172

**Published:** 2019-05-31

**Authors:** Dilawar Ahmad Mir, Krishnaswamy Balamurugan

**Affiliations:** Department of Biotechnology, Alagappa University, Karaikudi, India

**Keywords:** Cloning, PemK_Sa_ toxin protein, *C. elegans*, proteomics analysis, oxidative stress pathways, Western blotting, immune pathways, bioinformatic analysis

## Abstract

Bacterial exotoxins are major causative agents that infect by promoting cell and tissue damages through disabling the invading host immune system. However, the mode of action by which toxins modulate host immune system and lead cell death is still not completely understood. The nematode, *Caenorhabditis elegans* has been used as an attractive model host for toxicological studies. In this regard, the present study was undertaken to assess the impact of *Staphylococcus aureus* toxin (PemK) on the host *C. elegans* through global proteomics approach. Our proteomic data obtained through LC-MS/MS, subsequent bioinformatics and biochemical analyses revealed that in response to PemK_Sa_ a total of 601 proteins of *C. elegans* were differentially regulated in response to PemK_Sa_. The identified proteins were found to mainly participate in ATP generation, protein synthesis, lipid synthesis, cytoskeleton, heat shock proteins, innate immune defense, stress response, neuron degeneration, and muscle assembly. Current findings suggested that involvement of several regulatory proteins that appear to play a role in various molecular functions in combating PemK_Sa_ toxin-mediated microbial pathogenicity and/or host *C. elegans* immunity modulation. The results provided a preliminary view of the physiological and molecular response of a host toward a toxin and provided insight into highly complex host-toxin interactions.

## Introduction

*Caenorhabditis elegans* has a number of features that make it quite powerful model for biological studies and relevant to higher eukaryotes in areas such as genetics, cell death, neuroscience, aging, and development (Brenner, 1974; Horvitz, 2003; Sulston, 2003; Kenyon, 2005). The use of whole-organism for assays allows us to study the entire functional multicellular unit instead of a single cell (Kaletta and Hengartner, 2006). *C. elegans* has served as a successful model for assessing various neurotoxic chemicals (Boyd et al., 2003; Anderson et al., 2004; Melstrom and Williams, 2007; Rajini et al., 2008). Toxicological studies of *C. elegans* are likely relevant to higher eukaryotes. In addition, the ability to perform both forward and reverse genetic screens in *C. elegans* make it highly useful to understand host-toxin interactions at molecular levels (Brenner, 1974; Jorgensen and Mango, 2002).

In its natural environment, *C. elegans* interacts with a diverse range of microorganisms, including bacteria that can serve as food source. Certain soil bacteria have evolved their systems to fight against nematode predation. Some pathogenic bacteria are capable of proliferating in and killing the nematode by an infectious process or through the toxin secretions (Tan, 2002; Burlinson et al., 2013; Nandi et al., 2015). Bacterial pore-forming toxins are proteinaceous, that play a key role in pathogenesis (Huffman et al., 2004a; Aroian and Van Der Goot, 2007; Gonzalez et al., 2008) and constitute the single largest class of bacterial virulence factors, comprising 25–30% of all bacterial toxins (Alouf, 2003; Matarrese et al., 2007). *Bacillus thuringiensis* (Bt) coexists with *C. elegans* in its natural habitat. An important characteristic of Bt strains is to produce insecticidal crystal proteins (Cry) during sporulation stage. These proteins are highly specific and target insects and nematodes, and act as a viable alternative for agriculture pesticide (Pardo-Lopez et al., 2012). Several families of crystal proteins were observed to be toxic to *C. elegans*, such as Cry5, Cry6, Cry12, Cry14, Cry21, and Cry55 (Zhang et al., 2012; Yu et al., 2014).

Most of the bacterial toxins are targeting the mitochondria, which is a common strategy of pathogenesis in order to accomplish apoptosis (Matarrese et al., 2007). The mode of action of pore- forming toxins are to make holes in the cell plasma membrane, disrupting the membrane potential, ion balance, and breaching the cellular reliability which results in cell death or other faults, remarkably aiding in bacterial pathogenesis (Bravo et al., 2007)*. C. elegans* appears to be utilizing various defense responses against pathogens and harmful toxins through its innate immune system (Hasshoff et al., 2007) and behavioral defenses (Schulenburg and Müller, 2004). Previous studies have confirmed that *C. elegans* acquire a range of innate immune responses to protect itself when intoxicated by Bt toxic proteins such as activating p38 mitogen-activated protein kinase (MAPK) and c-Jun N-terminal-like kinase (JNK) pathway (Huffman et al., 2004b), triggering an unfolded protein response (Bischof et al., 2008), stress, and a hypoxic response (Bellier et al., 2009). *C. elegans* might use all innate immune responses in natural habitat, to protect from a diverse range of pathogens and their toxins. The *Pem* is a toxin-antitoxin loci of the R1/R100 plasmid (Bravo et al., 1987), and the term PemK_Sa_, is derived as follows: *Pem* indicates the loci, K represents killer protein and Sa represents *S. aureus* bacteria. The toxin (PemK_Sa_) is a sequence-specific endoribonuclease that recognizes the tetrad sequence UAUU. Data suggests PemK intoxication changes the translation of large pool of host genes; degrades the cellular RNA, limit the cell growth. The prolonged activation of PemK_Sa_ leads to cell death (Bukowski et al., 2013).

The endeavor of the current study is to identify the involvement of host proteins and their contribution during PemK_Sa_ toxin exposure. To gain insight, a proteomic analysis using nano-LC-MS/MS was performed to monitor the changes in protein regulation. The biological significance of this study is to identify system-wide effects of PemK_Sa_ intoxication on *C. elegans* at the protein level, growing the list of immune effectors potentially acting toward the bacterial toxins. This finding may help to understand the mechanism of bacterial pathogenesis during host-pathogen toxin interaction in other organisms.

## Materials and Methods

### Nematode *C. elegans* and Bacterial Strain Maintenance

Nematodes used in the present study were wild type N2 Bristol, and out-crossed mutant *ogt-1* (RB653) which were obtained from the CGC (Caenorhabditis Genetic Center, MN, USA). The animals were cultured as described earlier Stiernagle (2006). The plates were seeded with *Escherichia coli* OP50 strain, which was used as the food source for *C. elegans*. Eggs were isolated from mixed stage cultures of *C. elegans* grown on NGM plates using a simple bleaching protocol (5M KOH and bleach as 1:1 ratio) to grow synchronized L4 stage worms for the experiments.

### Preparation of *E. coli* BL21 DE3 Competent Cell

The synthetic pETDuet1-PemK_Sa_ vector was kindly provided by Dr. Benedykt Wladyka Malopolska, Center of Biotechnology, Gronostajowa Krakow, Poland. Transformation was performed as described by Sambrook and Russell (2006). Briefly, an overnight grown high-efficiency protein expression *E. coli* BL21 (DE3) cells were seeded into 20 mL of LB broth and incubated at 37°C with shaking speed (220–250 rpm) till the OD_600nm_ reaches 0.4–0.6. The culture was kept in ice for 30 min. After incubation, the cell pellet was collected using centrifugation at 3,000 × g for 10 min at 4°C. The supernatant was decanted and the pellet was suspended in 25 mL of ice cold 100 mM CaCl_2_ and centrifuged at 3,000 × g for 5 min. The pellet was dissolved in 500 μL of ice cold 100 mM CaCl_2_. Aliquots of 100 μL of BL21 (DE3) cell were dispersed into pre-chilled microfuge tubes, used for transformation.

### Protein Purification

Briefly, a single DE3 transformed colony was selected and inoculated overnight in 5 mL of LB media supplemented with ampicillin (100 mg/L), incubated at 37°C overnight for the expression analysis. later, 1 mL of mother culture was sub-cultured into 20 mL of LB media supplemented with ampicillin (100 mg/L) and incubated at 37°C till the growth reached mid-log phase (OD_600_ = 0.6). After reaching the required OD, the culture was induced by (0.1 mM) IPTG and incubated at 37°C with continuous shaking (200 rpm) for 4 h. After incubation, the cell pellets were collected using centrifugation and stored at −80°C for further analysis. Cells were lysed with an ultrasonication (Sonics & Materials, Danbury, CT, USA) in 1X PBS (NaCl 8 g, KCl 0.2 g, Na_2_HPO_4_ 1.44 g, and KH_2_PO_4_ 0.44 g, pH 7.5) with 5 μg/mL of DNase I, along with protease inhibitor cocktail and centrifuged at 10,000 × g for 20 min at 4°C to remove cell debris. Soluble recombinant proteins were affinity purified by using Ni-NTA His Bind Resin (Novagen 70666) as per the instructions. The PemK_Sa_ protein fractions was collected and stored at −80°C until use and purity of proteins was analyzed by gradient SDS-PAGE. The collected protein fractions were dialyzed by benzoylated dialysis tubing (Sigma Aldrich) as per manufacturer's protocol against 10 mM Tris-HCl, pH 7.4, and 150 mM NaCl buffer to remove the imidazole.

### Toxicity Assay

*Caenorhabditis elegans* intoxication assay was performed to determine the impact of toxin on nematode. Approximately, 20 L4 stage age-synchronized *C. elegans* were transferred from a lawn of *E. coli* OP50 to a 24-well plate containing *E. coli* DE3 (control), and transformed *E. coli* DE3 cells with and without IPTG induction (treated). Other experimental sets contain *E. coli* OP50 (control) or purified PemK_Sa_ toxin protein plus *E. coli* OP50 (treated), respectively. The plates were incubated at 20°C. *C. elegans* life span changes were monitored during the assays. The plates were scored for *C. elegans* viability after every 6 h. The worms that failed to respond to gentle touch using a worm picker were considered dead. All the experiments were carried out independently in biological triplicates. Kaplan–Meier survival analysis was used to compare the mean lifespan of control and treated nematodes and the error bars represent the mean ± SD (^*^p < 0.05).

### Morphological Assay

The age synchronized L4 stage *C. elegans* were exposed to 24 h with various concentrations (100 and 200 μg) of purified PemK_Sa._ After incubation worms were washed thoroughly with M9 buffer to take away the surface bound proteins. Morphological changes of *C. elegans* were examined using Light Microscope (Nikon).

### Protein Extraction and Sample Preparation for Mass Spectrometry

Based on the killing assay, final concentration of PemK_Sa_ toxin protein was maintained at 0.400 μg/mL and exposure time 24 h was chosen for subsequent translational studies. After toxin protein exposure, the *C. elegans* were washed thoroughly with M9 buffer to take away the surface bound toxin proteins. The washed animals were dissolved into 30 mM Tris-HCl buffer (pH 8.5 along with protease inhibitor cocktail). Subsequently, the solid homogenate was sonicated on ice for 3 min at 10 s pulse interval, debris was removed by centrifugation at 6,000 × g for 5 min and the resulting supernatant was collected. Protein concentration was determined by using Bradford reagent (Sigma Aldrich) (Bradford, 1976) and the protein concentration was maintained at 100 μg per sample. The collected samples were purified using 2D cleanup kit (GE Healthcare) per manufacturer's instructions. The protein profiling and quantification analysis has been done at out-sourcing center in Rajiv Gandhi Center for Biotechnology Kerala, India. Equal amounts of proteins from the control and treated *C. elegans* groups were subjected to in-solution trypsin digestion to make peptides. Prior to digestion the proteins samples were reduced by adding 5 μL of 100 mM DTT at 60°C for 30 min in the dark, alkylated by adding 5 μL of 200 mM iodoacetamide at 37°C for 30 min in the dark and vacuum dried for 30 min. Subsequently, the proteins were digested with trypsin (sequencing-grade modified trypsin, Sigma-Aldrich, USA; 1:25 w/w) in 50 mM ammonium bicarbonate buffer by incubating overnight at 37°C. The trypsinization was inhibited by adding 1 μL of formic acid for 20 min at 37°C. The digested peptides were centrifuged at 20,817 × g at 4°C for 15 min. The supernatants were collected and stored at −80°C for LC-MS/MS analysis. The peptides were analyzed by employing the two-dimensional (2D) nanoacquity UPLC^®^ system coupled with Quadrupole-time of flight (Q-TOF) mass spectrometer (SYNAPT-G2-HDMS, Waters, UK). Both systems were operated and controlled by MassLynx4.1 SCN781 software. The peptide level fractionation were performed using reverse phase column 1 at high pH (pH-8) in the first dimension, followed by RP column 2 at low pH (pH 2) in the second dimension.

### Mass Spectrometry Analysis

Peptides eluted from the nano-LC were subjected to mass spectrometric analysis on a SYNAPT^®^ G2 High Definition MS™ System (Waters, UK). The parameters were used as described in Dharmaprakash et al. (2014). The mass spectrometer was operated in the “resolution mode” with a resolving power of 18,000 FWHM and the data was obtained in “continuum” format. The data was acquired by quickly alternating between low energy functions and high energy function. The LC-MS data were analyzed using ProteinLynx Global SERVER™ v2.5.3 (Waters, UK) as per Gopinath et al. (2015) for protein identification as well as for the relative protein quantification. Noise reduction thresholds for high energy scan ion and low energy scan ion, peptide intensity were fixed at 150, 50, and 500 counts, respectively. The NCBI reference sequence database for *C. elegans* was used for search, throughout the database search; the protein false discovery rate was set to 4%. Work flow was planned in such a way that a protein was necessary to have at least one fragment ion matches and one peptide match, whereas a peptide was required to have at least three fragment ion match. Mass tolerance was set to 10 ppm for precursor ions and 20 ppm for fragment ions. Trypsin was selected as the primary digest reagent, used with a specificity of one missed cleavage. Oxidation of methionine was selected as the variable modification and carbamidomethylation of cysteine was selected as the fixed modification.

### Bioinformatics Analysis

High throughput protein profile and expression data were further subjected to bioinformatics analysis as per Schmidt et al. (2014). The gene ontology (GO) classification of differentially regulated proteins in this study was performed using the UniProt KB tool. Interaction among the differentially regulated proteins was performed using the STRING tool (Version 10.5) with a highest confidence score of 0.900. Functional annotation and gene enrichment scores of differentially regulated proteins were generated using the DAVID tool. The PANTHER tool was used to identify regulated protein class and pathways during toxin exposure (Myers et al., 2015; Villaveces et al., 2015).

### Detection of Oxidants and Antioxidants

The L4 stage *C. elegans* were exposed to *E. coli* OP50 and PemK_Sa_ toxin. Both exposed and control worms were washed several times thoroughly. Subsequently, the bacteria free worms were homogenized and protein concentration was kept at 100 μg/mL. Reactive oxygen species (ROS) was measured as per Scherz-Shouval et al. (2007) to study the level of ROS in host during PAO1 exposure. The H_2_O_2_ level in cell lysate supernatant was measured as described earlier (Wolff, 1994). SOD activity of *C. elegans* cell lysate supernatant (100 μg/mL of protein) was measured as per Paoletti and Mocali (1990). Catalase activity of *C. elegans* was measured as described earlier (Aebi, 1984). *C. elegans* carbonyl content was measured as per Levine et al. (1990). Each experiment was performed in biological triplicates and the error bars represent the mean ± SD (^*^p < 0.05).

### Lipid Peroxidation Quantification

Lipid peroxidation was determined as described earlier (Ohkawa et al., 1979). Briefly, the *C. elegans* cell lysate supernatant contained (100 μg/mL of protein) in a 50 mM phosphate buffer (pH 7.4) was mixed with 1:1 of ice-cold 10% TCA and centrifuged at 1,100 × g for 20 min. Five different concentrations (100–500 ng) of malondialdehyde (MDA) were used as standard. Equal volumes of 0.33% of 2-Thiobarbituric acid (TBARS) dissolved in 50% glacial acidic acid was added to the supernatant and the samples were mixed gently and boiled at 100°C for 20 min. After incubation, the samples were allowed to cool at room temperature and absorbance was measured at 532 nm.

### Extraction of *C. elegans* Lipids

*Caenorhabditis elegans* lipids were extracted as per Shi et al. (2016) with small modifications. Approximately, 10–12 large (10 cm) plates containing L4 stage *C. elegans* were washed from NGM plates, to a 24-well plate containing *E. coli* OP50 and PemK_Sa_ toxin for 24 hrs. After incubation, worms were washed thoroughly several times to remove the surface bound bacteria/ toxin proteins, and at −80°C. Lipids were extracted by adding 2 mL of ice-cold chloroform: methanol (1:1) to the frozen worm samples, mixed vigorously and incubated at −20°C overnight with occasional shaking. The following day, 400 μL of Hajra's solution (0.2 M H_3_PO_4_, 1M KCl) was added to each sample and mixed vigorously for a minute and centrifuged at 4,500 × g for minute which resulted separation of the organic and aqueous phase. The organic phase (lower chloroform phase) was removed and dried under CentriVap centrifugal vacuum concentrator and samples were directly used for Fourier transform infrared spectroscopic (FT-IR).

### Fourier Transform Infrared Spectroscopic Analysis

FT-IR spectroscopic analysis for *C. elegans* lipids was carried out as per described in Sheng et al. (2016) to validate the alterations or possible functional group change of the *C*. *elegans* lipid structure during the host-toxin interactions. The infrared spectra of isolated lipid of L4 stage *C. elegans* were recorded on FT-IR (Nicolet iS5, Thermo Scientific, USA) spectrometer. The spectra were obtained using potassium bromide (KBr) pellet technique. Briefly, potassium bromide (AR grade) was dried under vacuum at 45°C for 1 h, and 50 mg of KBr was added to 1 mg of sample to prepare KBr pellet. The spectra were scanned in the 4,000–400 cm^−1^ range. All the IR spectra were plotted as absorbance units, vs. wave number using Origin software (JebaMercy et al., 2013).

### Western Blotting

The protein content of whole cell extracts (at different time points of exposure) was prepared using 1X PBS buffer along with protease inhibitor cocktail. Protein concentration was determined by the Bradford assay. For each time point, 100–200 μg of total protein was boiled in 5X Laemmli's buffer for 3 min followed by short spin at 3,000 × g. The protein samples were subjected to 12% SDS-PAGE, followed by transfer on nitrocellulose membranes as described earlier (Durai et al., 2014). After transfer, the membranes were exposed to primary antibodies overnight at 4°C on a shaker. Before and after incubation with secondary antibodies the membranes were washed extensively using alkaline phosphate-buffer (TBS) containing Tween. The binding of antibody was detected by enhanced chemiluminescence containing substrate nitro-blue tetrazolium chloride and 5-bromo-4- chloro-3-indolyphosphate. The antibodies used in the present study were purchased from Santa Cruz Biotechnology, Inc. PDI [sc-20132], JNK-1[sc-571], p38 [sc-17852], HSF-1[sc-9144], HSF-90 [sc-1055], SGK-1 [sc-33774] RACK-1[17754], Caspases-3 [sc-7148], and beta-actin purified mouse immunoglobulin (A1978) purchased form (Sigma-Aldrich) working concentration was kept at 1:1,000–1:2,000.

### Statistical Analysis

All experiments were performed independently in triplicate. The statistical significance of data was analyzed by -one way ANOVA and Duncan test (SPSS Chicago, IL, USA) at significance level of *p* < 0.05.

## Results

### Plasmid pETDuet1-PemK_Sa_ Expression

**Figure 1:**
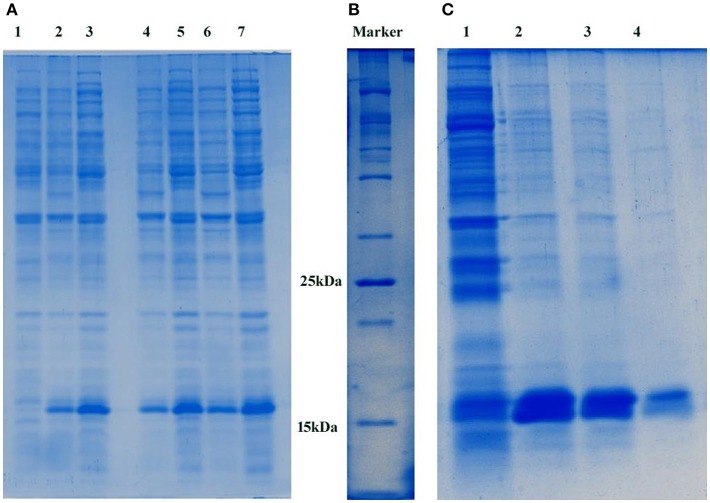
**(A)** Expression of PemK_Sa_ toxin protein. Lane 1 contains *E. coli* DE3 total cell lysate protein, Lanes 2, 4, and 6 contains uninduced total cell lysate protein of *E. coli* DE3. Lanes 3, 5, and 7 contains IPTG induced total cell lysate protein of *E. coli* DE3. Concentration: Lanes 1, 2, and 3 (50 μg) Lanes 4 and 5 (75 μg) and Lanes 6 and 7 (100 μg), respectively. **(B)** 100 kDa protein marker **(C)** Molecular weight corresponding to PemK_Sa_ is ~16 kDa. PemK_Sa_ protein was purified affinity binding analyzed by 12% SDS-PAGE. Lane 2, 3, and 4 contains purified elute fractions of PemK_Sa_ protein.

The pETDuet1-PemK_Sa_ plasmid which encodes a toxin protein (PemK_Sa_; 112 amino acids) was transformed into *E. coli* DE3 cells, the desired recombinant cells are provided in Figure S1A. Plasmid DNA was extracted, and the purity of the plasmid was analyzed in 1% agarose gel as provided in Figure S1B. The extracted pETDuet1-PemK_Sa_ plasmid DNA was extracted (molecular weight of 5.5 kb) (Supplementary Figure 1C) and double digested using restriction enzyme (*EcoR1* and *Sac1*). Purity of the digested plasmid was checked, a DNA bands were observed in the agarose gel with an approximate molecular weight of 1.5 kb and 750 bp, respectively (Supplementary Figure 1D). Upon induction with IPTG, *E. coli* DE3 cells carrying vector (pETDuet1-PemK_Sa_) expressed large quantities of tagged His6-PemK_Sa_ protein, as evidenced by gradient SDS**-**PAGE. Approximately, the visualized protein band with an approximate molecular weight 16 kDa is PemK_Sa_ protein as provided in Figure 1A. The highest yields were obtained between 4 and 5 h after induction by 1 μL/1 mL of IPTG at 37°C temperature. Figure 1B is a 100 kDa protein marker. Un-induced culture showed some leaky expression of PemK_Sa_ toxin protein, whereas the culture induced by IPTG expressed the PemK_Sa_ protein in bulk quantity. The PemK_Sa_ toxin was purified per manufacturers protocol described under experimental procedures and purified elution fraction was analyzed by using the 12% SDS-PAGE as provided in Figure 1C.

### Killing Assay

**Figure 2:**
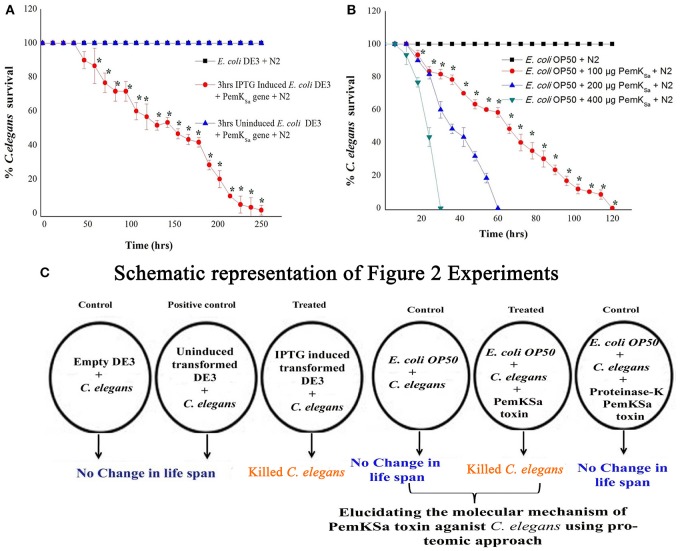
Physiological assays showing the impact of PemK_Sa_ toxin proteins on *C. elegans*. **(A)** In liquid killing assays, IPTG induced *E. coli* DE3 significantly (*p* < 0.05) killed *C. elegans* at 250 ± 10 hrs. **(B)** In liquid killing assays, purified PemK_Sa_ toxin protein fractions caused complete killing of *C. elegans* concentration at 200 and 400 μg/mL killed *C. elegans* at 30 and 60 h, respectively. PemK_Sa_ protein concentration maintained at 400 μg/mL treated overnight with Proteinase-K enzyme at 37°C has not killed the nematode, which ensured death was due to protein only. **(C)** A graphically representation of the outcome of Figure 2. Each experiment was performed in triplicates. ^*^Differences were considered significant at *p* < 0.05.

To evaluate the intoxication effect of a PemK_Sa_ toxin protein on bacterial-feeding nematode a liquid killing assay was performed *C. elegans* fed with IPTG induced *E. coli* DE3 (transformed) strain intoxicated the nematodes after exposure. In other side, *C. elegans* fed on wild type *E. coli* DE3 as well as with IPTG un-induced transformed cells not led mortality or any other adverse affect to nematodes. Quantitative result of this killing assay is graphically represented in Figure 2. In almost all cases where intoxication was seen, the nematodes were died within a time frame. However, IPTG induced DE3 PemK_Sa_ required 250 ± 10 h (*p* < 0.05) for the complete killing of *C. elegans* (Figure 2A). Killing assay time points revealed that the continuous exposure of *C. elegans* with 3 h IPTG induced *E. coli* DE3 in liquid media led to a significant (*p* < 0.05) decrease in the life span of nematodes.

To determine the impact of PemK_Sa_ toxin on host, the nematode toxicity assay was performed with purified PemK_Sa_ toxin protein and killing ability of PemK_Sa_ on *C. elegans* were tested. In liquid killing assay, the N2 worms exposed with PemK_Sa_ toxin led to a significant (*p* < 0.05) decrease in the life span of nematodes. PemK_Sa_ required 30 ± 2 h (*p* < 0.05) for the complete killing of *C. elegans* (Figure 2B). For LT_50_, (time for half to die) it required 15 ± 3 h. N2 *C. elegans* grown on control bacteria (worms in *E. coli* OP50) displayed a normal life span. Liquid killing assay revealed that the continuous exposure of *C. elegans* with PemK_Sa_ toxin causes mortality. The survival percentages of *C. elegans* in the presence of PemK_Sa_ toxin were significantly lower (*p* < 0.05) compared with the *E. coli* OP50. The purified PemK_Sa_ toxin protein completely killed *C. elegans* on 30, 60, and 120 h at the concentrations of 400, 200, and 100 μg, respectively. The PemK_Sa_, toxin proteins fractions treated with proteinase-K overnight at 37°C showed no significant difference in the lifespan between control and worms exposed with proteinase-K treated PemK_Sa_ toxin protein fractions (Figure 2B). It suggested that the toxin protein fractions modulated the *C. elegans* lifespan and morphology after exposure. Each experiment was performed in biological triplicates and the error bars represent the mean ± SD (^*^p < 0.05).

### Global Proteome Response of *C. elegans* Upon PemK_Sa_ Toxin Exposure

The total proteome of control and treated N2 *C. elegans* were initially analyzed using the SDS-PAGE in which prominent differential regulated protein bands were identified and listed as provided in Figure S2. The results of the killing assay and differential protein pattern of *C. elegans* on SDS-PAGE have lead us to investigate the primary molecular mechanism that have caused *C. elegans* mortality through proteomic approach using liquid chromatography tandem mass spectrometry (LC-MS/MS) to identify the proteins involved in *C. elegans* mortality and other physiological changes. Based on the post-MS data protein identification and expression analysis, a total of 601 differential regulated proteins were detected. The relative expression ratio of downregulated and upregulated proteins between control and treated samples was fixed as ≥-1.5 and ≥1.5, respectively, of all the biological replicates (*p* < 0.05). Among the 601 differentially regulated proteins 361 and 240 proteins were found to be downregulated and upregulated, respectively. The list of differentially regulated proteins is provided in Tables S1, S2. High throughput protein profile and expression data were further subjected to bioinformatic analysis.

### Gene Ontology Annotation for Differentially Regulated Proteins

**Figure 3:**
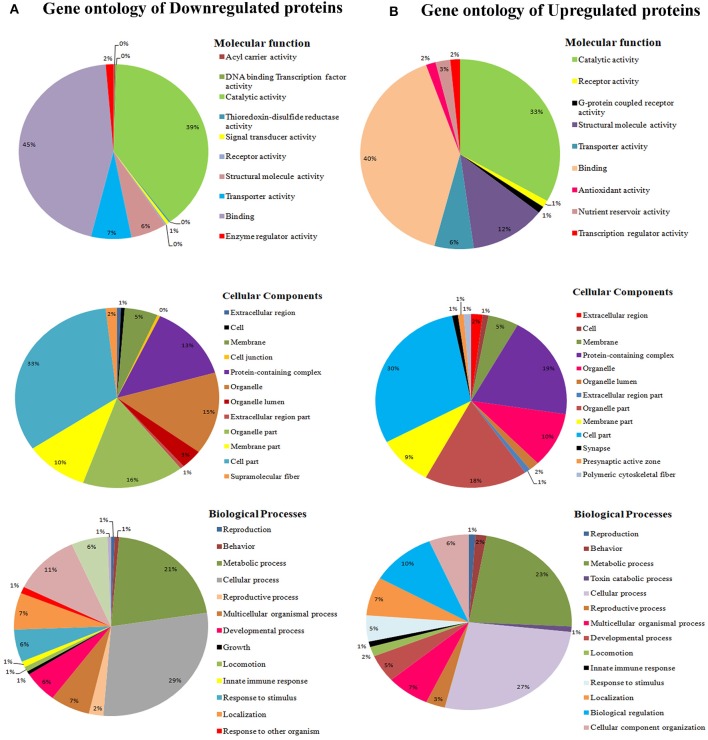
**(A)** Gene ontology analysis using the UniProtKB online tool showed downregulated proteins are involved in binding activity, catalytic activity, cell parts, cellular processes, single organism process, metabolic processes locomotion, oxidative stress, and developmental processes. **(B)** Whereas, *C. elegans* upregulated proteins are involved catalytic activity, receptor activity, cell part, macromolecular complex, and metabolic processes.

To classify the regulated proteins of *C. elegans* exposed to PemK_Sa_ toxin into potential physiological functions. A Gene ontology (GO) annotation and functional enrichment analysis was carried out using the UniProtKB tool. The differentially regulated proteins were categorized into molecular functions, biological processes and cellular components. According to the GO functional annotation, the largest set of downregulated and upregulated proteins belongs to a functional groups involved in binding activity, catalytic activity and structural molecule activity. The predominant biological processes of differentially regulated proteins are involved in the metabolic process and cellular process as shown in Figures 3A,B. Several identified regulated proteins are involved in Na^2+^ and Ca^2+^ voltage gated channels which lead to degradation in the synapses of neurons and immune response pathway.

**Figure 4:**
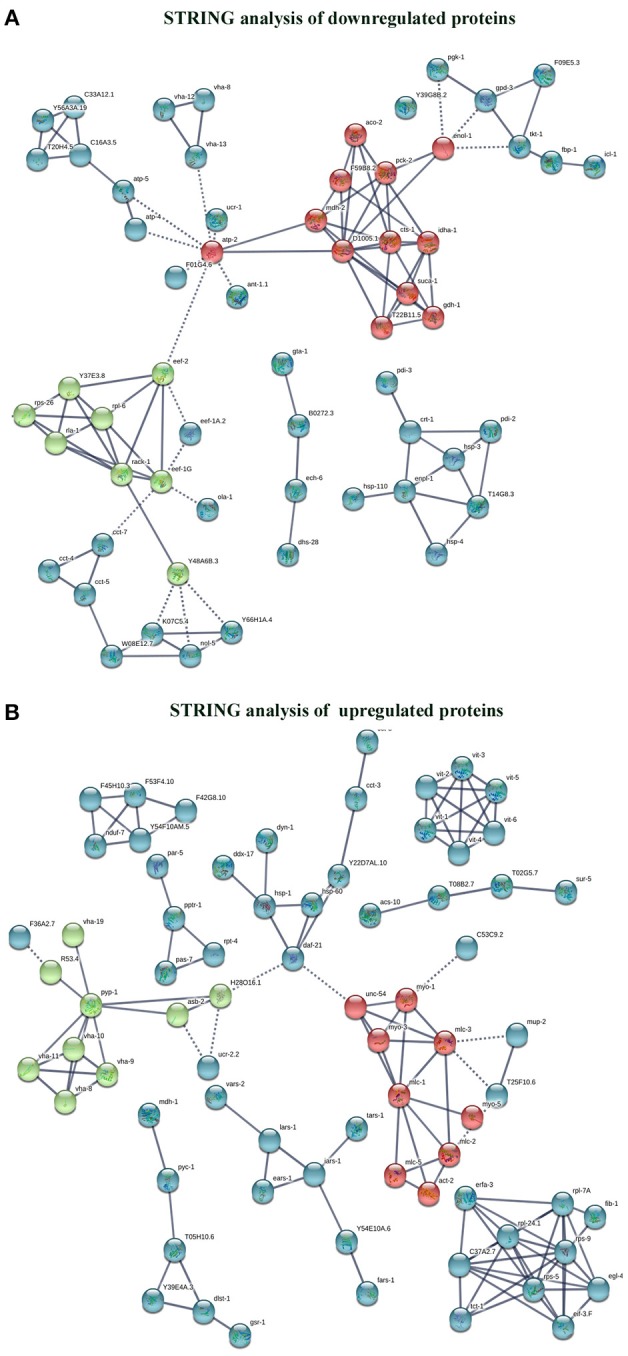
Interactome map among regulated proteins was performed using STRING tool with a highest confidence threshold score (0.900). The interaction map showed link between identified regulated proteins of *C. elegans*. **(A)** The downregulated proteins showed interaction among translation, ATP biosynthesis, ETC chain, chaperon, cytochrome c family, nucleotide binding, and oxidation-reduction responsible proteins. **(B)** Whereas, upregulated proteins showed interaction between HSP, stress, muscle, mitochondrial, and ribosomal proteins.

The interactome network using STRING tool was built separately for both downregulated and upregulated proteins to find out the interaction among regulated proteins and predicted the functional association. The interaction map of downregulated proteins displayed the relationship between molecular players involved in translation, ATP biosynthesis and metabolism, ETC chain, chaperon, adult life span, ribosome biogenesis, cytochrome family and oxidation-reduction processes (Figure 4A). The proteins associated with these processes either directly interacting with each other, or interact through their partners. The potential deregulation pathways connected with PemK_Sa_ intoxication was identified using the STRING and PANTHER tools. The pathways mostly downregulated by PemK_Sa_ toxin are presented in Table 1. The STRING analysis of upregulated proteins show interaction between HSP proteins with PDI-6 (stress proteins), ATP synthetic subunits (H28016.1), ribosomal proteins (RPL-2), and MYO-3 and UNC-54 (muscle proteins), presented in Figure 4B. Nicotinic acetylcholine receptor signaling pathway is the major upregulated pathway connected with PemK_Sa_ intoxication in *C. elegans* presented in Table 1. Protein classes mostly affected by PemK_Sa_ toxin are presented in Table 2.

**Table 1:** Several pathways regulated in *C. elegans* during PemK_Sa_ toxin protein exposure were identified using online PANTHER tool [downregulated (D); upregulated (U)].

**S. No**	**PANTHER pathways**	**Expected**	**Fold enrichment**	**Raw *P-*value**	**FDR**
1	ATP synthesis (D)	0.11	27.64	3.91E-04	7.42E-03
2	Pyruvate metabolism (D)	0.16	25.80	4.73E-05	1.80E-03
3	TCA cycle (D)	0.12	24.18	5.31E-04	8.97E-03
4	Glycolysis (D)	0.17	23.45	6.37E-05	1.61E-03
5	Parkinson disease (D)	0.82	12.17	3.73E-08	2.83E-06
6	Cytoskeletal regulation by Rho GTPase (D)	0.48	10.40	2.05E-04	4.46E-03
7	Ubiquitin proteasome pathway (D)	0.60	9.92	5.96E-05	1.81E-03
8	Apoptosis signaling pathway (D)	0.47	8.60	1.72E-03	2.62E-02
9	Huntington disease (D)	0.98	8.19	1.25E-05	6.32E-04
10	Nicotinic acetylcholine receptor signaling pathway (U)	0.67	8.97	8.13E-05	6.18E-03

**Table 2:** PemK_Sa_ intoxication has regulated several protein classes identified using online PANTHER tool.

**PANTHER protein class**	**Expected**	**Fold enrichment**	**Raw *P*-value**	**FDR**
Storage protein	0.07	87.46	9.83E-10	3.87E-08
Chaperonin	0.09	34.98	1.62E-04	2.46E-03
Chaperone	0.38	13.25	5.91E-05	1.16E-03
Aminoacyl-tRNA synthetase	0.17	23.32	4.70E-05	1.03E-03
Ligase	1.54	5.83	3.56E-05	8.76E-04
ATP synthase	0.23	17.28	1.33E-04	2.18E-03
Cation transporter	1.01	5.93	6.76E-04	8.32E-03
Ribosomal protein	1.08	16.66	3.17E-16	3.12E-14
RNA binding protein	4.12	6.06	1.32E-12	8.68E-11
Nucleic acid binding	8.22	3.77	2.67E-10	1.31E-08
Dehydrogenase	1.44	7.64	3.93E-07	1.11E-05
Oxidoreductase	3.39	5.02	9.01E-08	2.96E-06
Translation factor	0.58	6.86	3.31E-03	3.84E-02
Actin family cytoskeletal protein	1.04	6.75	1.13E-04	2.03E-03
Cytoskeletal protein	2.27	3.96	5.79E-04	7.61E-03
Hydrolase	8.20	2.44	2.46E-04	3.47E-03

### Functional Annotation and Gene Enrichment Score

**Figure 5:**
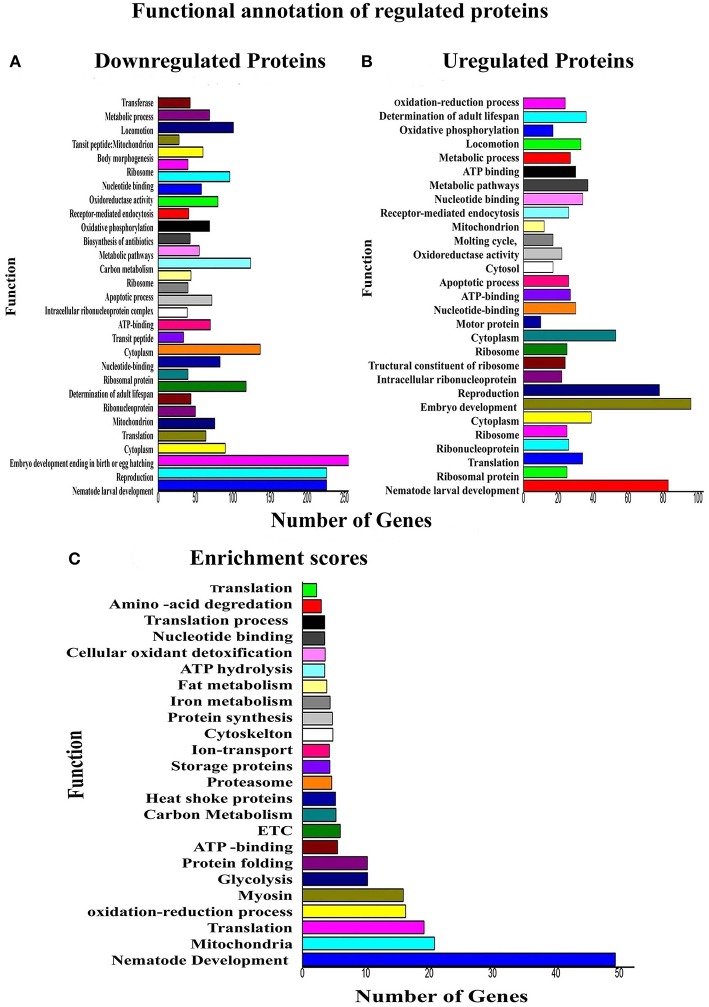
Functional annotations and protein enrichment scores of regulated proteins was performed using the DAVID tool. **(A)** The biological functions which have more number of downregulated proteins in response to PemK_Sa_ toxin exposure were embryo development; nematode development and reproduction. **(B)** Biological Functions which contains more number of upregulated proteins against toxin exposure is embryo development. **(C)** Physiological function having highest protein enrichment scores is nematode development and mitochondrial functions.

Regarding the functional annotation reflects the nature of biology, whereby one protein could play multiple roles in different biological processes. Functional annotation of downregulated [*N* = 361] and upregulated [*N* = 240] proteins was performed using the DAVID tool. Physiological functions that are highly affected in response to PemK_Sa_ toxin proteins exposure are embryo development; reproduction, nematode development and cytoplasm are presented in Figures 5A, B. The biological functions that have the highest protein enrichment scores of differentially regulated proteins in response to PemK_Sa_ exposure are nematode development, mitochondrion, translation, and oxidation reduction processes are presented in Figure 5C.

### PemK_Sa_ Toxin Affected Nematodes Intestine

**Figure 6:**
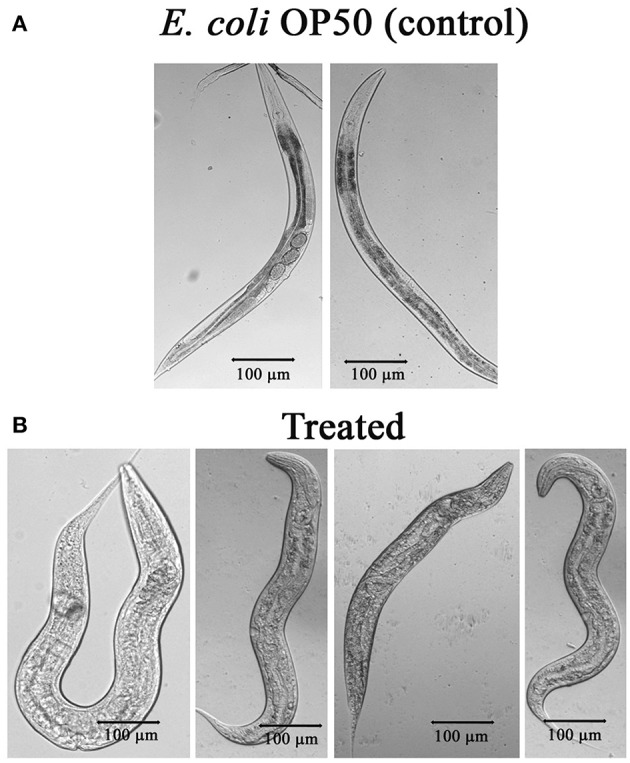
**(A)** Microscopic images of *C. elegans* exposed with *E. coli* OP50. **(B)** *C. elegans* exposed with PemK_Sa_ toxin (concentration 100–200 μg/mL) showed full swollen body morphology, damaged and disintegrated underlying inner body tissues.

The mechanism of toxicity of Bt crystal proteins toward insects is accepted that it damages the gut of nematode (Wei et al., 2003). To study the impact of the PemK_Sa_ intoxication on nematode morphology, microscopic images [Nikon Eclipse Ti-S, Japan] of toxin exposed *C. elegans* were analyzed. Size of the worms was determined and compared by taking the photographs. The results showed, the intoxicated worms have full swollen body, damaged and disintegrated underlying inner body tissues, and digestive track (Figure 6). This microscopic data representing the morphological defects in *C. elegans* during toxin exposure are corroborated with the mass spectrometric data where the downregulation of muscle and cuticle collagen related genes (*dpy-18, ifb-1, mua-6, unc-15, myo-2, act-1, dim-1, tba-2, cct-1, cutl-5, tba-1, tba-8, pfn-1*, and *pfn-2*) was ascertained.

### Regulation of Oxidant and Antioxidant Proteins of *C. elegans*

**Figure 7:**
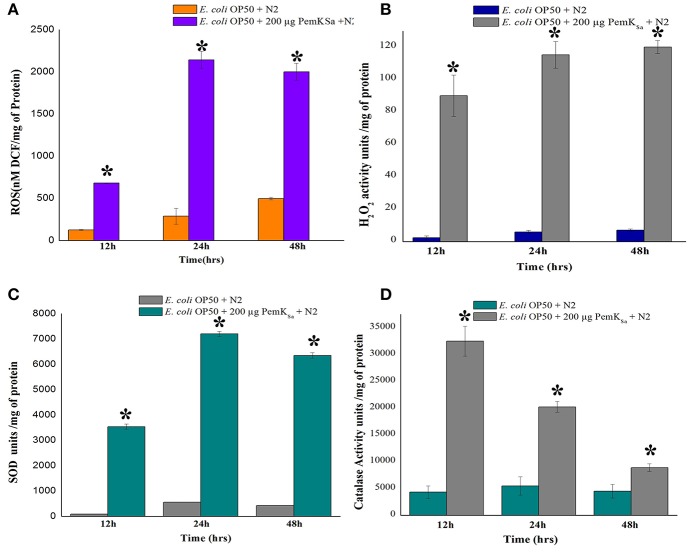
Quantitative analysis of oxidant and antioxidant proteins of control and treated *C. elegans*. **(A)** ROS estimation, **(B)** H_2_O_2_ estimation, **(C)** Quantification of SOD activity, and **(D)** Quantification of catalase activity. Data were expressed as mean value of three experiments and the error bars represent Mean ± SD (^*^p < 0.05).

Several stress and chaperones proteins (PDI-2, ENPL, HSPs, PDI-1/2/3, SODH-1, CCT-1/2/3/7, TRX-4, DAF-21, GST-5/36, CDC-48.1, IIF-2, KAT-1, and RNP-6) were identified to be differentially regulated during PemK_Sa_ exposure to *C. elegans*. This result lead us to suspect whether expression of oxidant proteins in nematode during intoxication have induced the generation of ROS as a protective measurement. To investigate this, we determined the levels of ROS using 2′, 7′-dichlorodihydrofluorescein diacetate (H_2_DCFDA) staining. ROS induction was examined at three-time points (12, 24, and 48 h), it was found that ROS generation was higher in worms exposed to PemK_Sa_ toxin compared with that of control (fed with OP50 food source) as presented in Figure 7A. To assess the hydrogen peroxide level of the PemK_Sa_ toxin exposed *C. elegans* at three time points (12, 24, and 48 h). It was found that H_2_O_2_ generation was significantly higher in exposed samples compared with that of control worms, for all the three time points as presented in Figure 7B.

The antioxidant enzymatic proteins serve as a key factor to maintain the redox homeostasis. In this study the expression of antioxidant proteins viz, glutathione reductase (GSR-1), peroxiredoxin (PRDX-3), probable glutathione S-transferase 5/36 (GST-5/36), superoxide dismutase enzyme (SOD), peroxidise (SKPO-1), thioredoxin (TRX) have corroborated well with the *in vivo* detection of hydrogen peroxide (H_2_O_2_) associated ROS generation which directly leads to the accumulation of molecular damage. The expression levels of antioxidant enzymes (SOD and CTL) were also measured to determine the extent to which increased antioxidant defenses could account for the observed levels of oxidative damage in L4 stage worms. The estimation of SOD was evaluated in both control and treated samples at three different time points (12, 24, and 48 h). The measurement of SOD alone showed significant increase of 10-fold in treated L4 stage animals, compared with that of control as presented in Figure 7C. SOD acts as a scavenger for toxic superoxide radical, which is concerned in lipid peroxidation. The significantly increased activity of SOD may be due to compensatory efforts generated by SOD to combat oxidative stress. Quantitative spectrophotometric analysis of catalase activity of L4 stage nematodes exposed to PemK_Sa_ toxin protein showed significantly high catalase activity at 12 and 24 h time points compared with that of control fed with *E. coli* OP50 as presented in Figure 7D. The mean activity values of catalase enzyme were decreased in treated samples at 48 h. The decreased activity of catalase at 48 h treated samples compared with that of controls, suggested the rescue against hydrogen peroxide free radicals and other oxidative stresses appeared to be reduced in *C. elegans*.

### Estimation of *C. elegans* Protein Carbonyl Content

**Figure 8:**
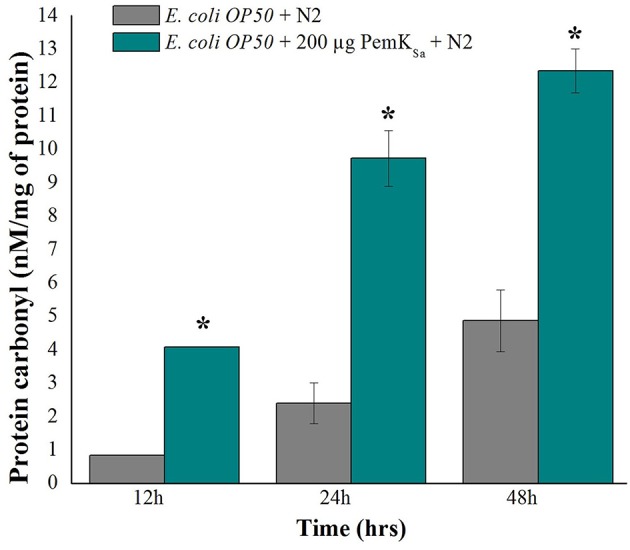
Protein carbonyls content quantification of control and PemK_Sa_ treated *C. elegans* for three time points (12, 24, and 48 h) showed significant low carbonyl content in control fed with *E. coli* OP50. Data were expressed as mean value of three experiments and the error bars represent SD ± mean (^*^p < 0.05).

The protein carbonyl content is a molecular marker to test SOD. The protein carbonyl contents were evaluated in both control and treated samples at three different time points (12, 24, and 48 h) by 2, 4-dinitrophenyl hydrazine. Worms exposed with PemK_Sa_ toxin protein extensively showed an enhanced level of protein carbonyl content 12.3 ± 1.20 nM/mg compared with that of the control *C. elegans* as presented in Figure 8. Prominent high levels of protein carbonyls in treated sample could be caused by a decrease in the turnover rate of the oxidized proteins or by an increase in protein oxidation. In the present study, a number of identified proteins appear to be responsible for amino acid degradation and some marker proteins were regulated in this study which plays a key role to degrade (proteasome activity) the oxidized proteins.

### PemK_Sa_ Toxin Regulated the Lipid Metabolism Related Proteins

**Figure 9:**
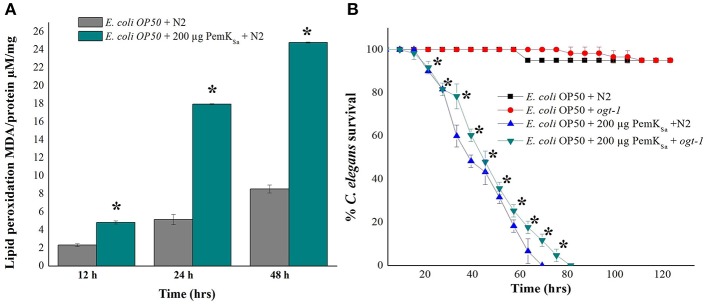
**(A)**. Lipid peroxidation quantification of control and treated *C. elegans* for three time points (12, 24, and 48 h) showed high lipid peroxidation in toxin exposed *C. elegans* samples compared with that of control. **(B)** In liquid killing assay PemK_Sa_ toxin protein exposed *ogt-1* mutant worm showed significant (*p* < 0.05) more survival rate 80 ± 5 h compared with that of wild type N2 worms. However, the mutant and wild type N2 worms fed with *E. coli* OP50 showed normal life. Data were expressed as mean value of three experiments and the error bars represent SD ± mean (^*^p < 0.05).

Lipid metabolism related proteins which were identified to be regulated in this study include: ADS-1, DHS-28, NPA-1, POD-2, ACBP-1, AAGR-4, PCK-1, TPI-1, T25B9.9, DLAT-1, LBP-6, NMT-1, ALH-9, IBP-6, RIBO-1, SUP-46, FBP-1, FAT-6, PCK-1/2, ADS-1, PGK-1, GSY-1, ACS-11, and Y37D8A.2. Regulation of these proteins indicated an alteration of fat storage molecules in living organism. To validate the fat and lipid deposition change in exposed *C. elegans*, lipid peroxidation assay was performed. Quantifying a lipid peroxidation is an effective way to measure the effect of oxidative damage. Concurrent estimation of *C. elegans* lipid peroxidation for both control and treated samples at three time points (12, 24, and 48 h) showed the significantly higher level of Thiobarbituric acid (TBARS) formation by H_2_O_2_ induced lipid peroxidation in the treated *C. elegans* samples compared with that of control as presented in Figure 9A*. C. elegans ogt-1* mutant encodes truncated O-linked N-acetylglucosamine proteins that lacks catalytic activity to promote glycosylation as well as the storage of fats and lipid. In liquid a killing assay, toxin protein exposed *ogt-1* mutant worms showed complete mortality at 75 ± 5 h, where the mean life span of *ogt-1* mutant is higher than mean life span of wild type N2 worms exposed to toxin proteins. On the other hand, the wild type N2 and mutant *ogt-1* worms fed with *E. coli* OP50 food source showed zero mortality as presented in Figure 9B.

### FT-IR Analysis of *C. elegans* Lipids

**Figure 10:**
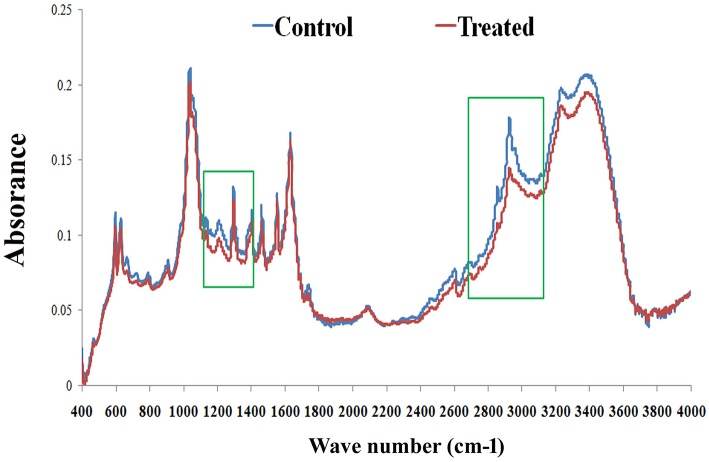
FT-IR analysis of *C. elegans* lipids. The representative FT-IR spectra of lipids (control and treated) in the 4,000–400 cm^−1^ region. However, the average FT-IR spectra of the control and treated showed major signature peak difference at 3,000–2,800 cm^−1^ (fatty acids). Data were expressed as mean value of three experiments and the error bars represent SD ± mean (^*^p < 0.05).

FT-IR analysis was carried out to examine the effect of PemK_Sa_ treatment on *C. elegans* lipids. FT-IR has been used as a potent tool to study biomolecular complex structures (Stuart, 2012). The FT-IR analysis of *C. elegans* fatty acids showed prominent intensity peak differences between the control and treated samples (Figure 10). The results showed major signature peak difference at 3,000–2,800 cm^−1^ corresponding to fatty acids (Helm et al., 1991), compared to control. The treated sample showed lower intensity at 3,100 cm^−1^ (N-H stretching of proteins attached with lipids) (Akkas et al., 2007), 2,930 cm^−1^ (CH_2_ anti-symmetric stretch of lipids), 2,870 cm^−1^ (CH_3_ symmetric stretching of lipids) (Melin et al., 2000; Toyran and Severcan, 2003), 2,450 cm^−1^ (C-O-C asymmetric stretching of glycogen and nucleic acids) C = O cm^−1^ (stretching of phospholipids compared with that of control), and 1,250–1,150 cm^−1^(PO_2_ asymmetric stretching of phospholipids). It evidenced that the spectral differences were observed to be in the *C. elegans* fatty acids region. These findings suggested that the impact of toxin protein on fat molecules in a host system. Variation in the FT-IR spectra of toxin treated sample has corroborated the results of LC-MS/MS and lipid peroxidation assay.

### PemK_Sa_ Toxin Regulated Innate Immunity Proteins Validated by Western Blotting

**Figure 11:**
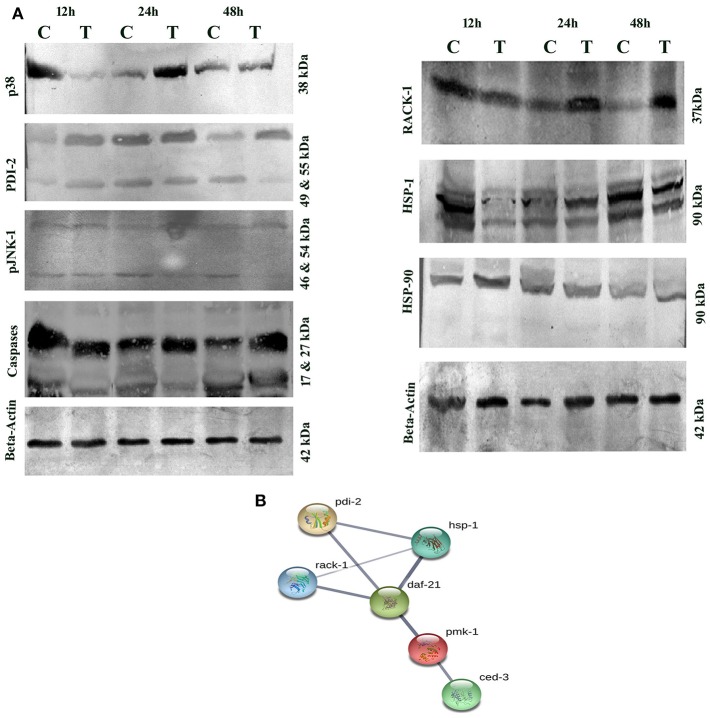
**(A)** Western blotting analysis of *C. elegans* JNK and MAP Kinase pathway specific proteins (p38, PDI-2, pJNK, caspases, RACK-1, HSP-1/90, and SGK) were performed. *C. elegans* exposed with *E. coli* OP50 and toxin proteins at 12, 24, and 48 h a pathway specific proteins were detected using the specific antibodies. The protein expression level at each time point was normalized to that of β-actin. PDI-2 is stress responsible protein which is found to assist in protein folding. **(B)**. UniportKB protein-protein interaction showed close association and interaction between PMK-1(p38), RACK-1, PDI-2, HSF-1, and HSF-4 Proteins.

Several host immune-responses (JNK and MAP Kinase pathways) proteins were differentially regulated and validation analysis of these proteins was performed through Western blotting analysis. Inadequate or excessive expressions of JNK and MAP Kinase pathway specific proteins have adverse immune response consequences. The expression patterns of p38 and JNK-MAPK pathway coupled proteins through Western blotting analysis showed high abundance of p38 upto 24 h and low abundance at 48 h. Beta-actin was used as the internal control as presented in Figure 11A. During host-toxin interaction several stress proteins were regulated [for example, PDI-1/2/36/ CRT-1, TPI-1, NHR-57, CST-1, CBS-1, GSR-1, GST-5/36, PRDX-3, PPTRA-1, KAT-1, SODH-1]. PDI-2 encodes disulfide isomerase, assist in protein folding and stress. The PDI-2 in particular, was detected and monitored by immunoblotting. The study showed high abundance of PDI-2 in treated sample compared with that of control. The Western blot results clearly supported the differential expression of PDI-2 during toxin exposure as presented in Figure 11A. PDI-2 results showed that the relative fold change of three isoforms of PDI-2 protein in worms during exposure with PemK_Sa_ toxin in a time course manner. PDI-2 protein regulates expression of the HSP-4 and HSP-1. RACK protein showed upregulation, whereas HSP-90 showed downregulation. The isoform of pJNK-1 protein (46 kDa) was downregulated at 12 and 24 h, whereas protein had diminished as the exposure time of PemK_Sa_ toxin was exceeded upto 48 h.

**Figure 12:**
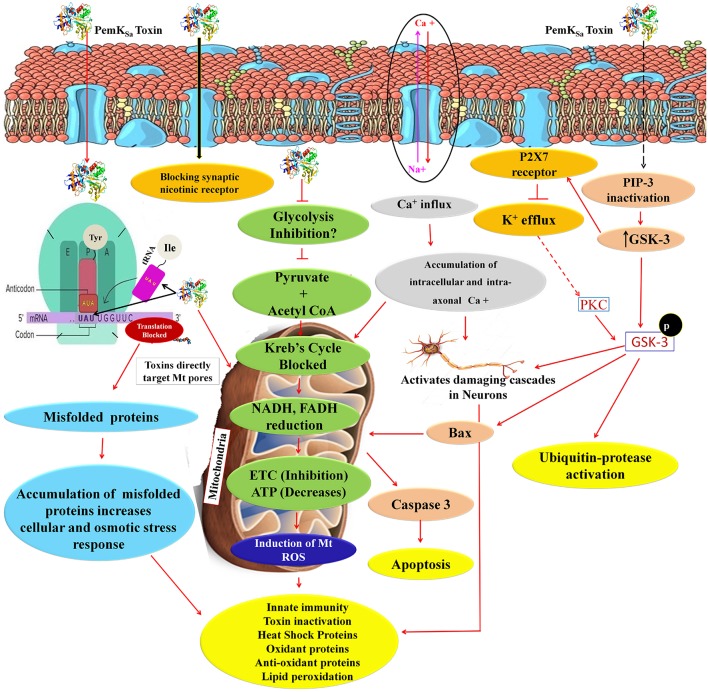
An overview of proteins and pathways activated and targeted by PemK_Sa_ toxin in *C. elegans*. PemK_Sa_ is sequence-specific endoribonuclease recognizing the tetrad sequence UAUU mRNA and cut it between U and A nucleotides. Chances are that PemK_Sa_ in present study might targeted the codon of Tyrosine and anticodon of Isoleucine UAU, it either degrade the mRNA or stopped the protein synthesis, or generate mutated proteins which lack the native confirmation. Toxins are directly targeting the host cell mitochondria and blocks the voltage gated channels and other important pores. Inhibition of glycolysis will also decrease coenzyme NADH and FADH which act as reduced powers and inhibit the ETC. PemK_Sa_ might be blocking synaptic nicotinic receptor and affect or degenerate the *C. elegans* neurons. Phosphatidylinositol 3,4,5*-*trisphosphate (PIP3), is a component of cell membrane that activates numerous signaling pathways resulting in cell proliferation, growth, survival, glucose transport and protein synthesis. Glycogen synthase kinase-3 (GSK-3), a serine-threonine kinase, is an important component of the glycogen metabolism pathway. It is highly expressed in neurons for development and repair which activates ubiquitin-protease for degradation. GSK-3 inhibiting phase II gene expression in the oxidative stress defense. Inhibition or blockage of voltage gated Na^2+^, Ca^2+^, and K^2+^ channels play a critical role in the generation and propagation of action potentials in neurons and muscle cells. Protein kinase C (PKC-1) phosphorylates a range of cellular proteins. Which acts as an extracellular signal regulated mitogen-activated protein kinase (ERK/MAPK), in response to diverse sensory neurons. Its role in regulation differs depending on the neuron in which it is acting. Required for incomplete resistance to antimitotic toxins. In *C. elegans* an increased ROS, H_2_O_2_ and expression of other oxidant proteins activates innate immunity and anti-oxidant proteins for toxin inactivation.

Altered metabolic process and the extent of an oxidative stress induce native apoptotic pathways. Several proteins responsible for phagocytosis and apoptosis (NDK-1, TFG-1, CDKR-1 ASP-1, CCM-3, IFF-2, CAR-1, ICD-1, DYN-1, and ARF-1.2) were found to be regulated in our study. The caspases-3 (CED-3) in particular, was detected and monitored by immunoblotting and p38 (PMK-1) protein showed interaction with CED-3 as presented in Figure 11B. The study showed upregulation of CED-3 protein (active caspases) in treated samples compared with that of the controls. The molecular weight of two active caspases isoforms are 17 and 27 kDa, respectively, as presented in Figure 11A. An overview of proteins and pathways activated and targeted by the PemK_Sa_ toxin in *C. elegans* are presented in Figure 12.

## Discussion

In this study, we have assessed the impact of *S. aureus* PemK toxins on the *C. elegans*. The studies have revealed toxin does not merely affect the nematode lifespan/ survival and fertility and survival, but also causes significant damage to the nematode intestine and fat storage. In survival assays, *E. coli* DE3 expressing PemK_Sa_ toxin protein caused significant mortality in *C. elegans*. We have also analyzed the *C. elegans* proteome to decipher the key differentially regulated players responsible for mediating virulent toxin determinants. A unique aspect in our experimental setup is constant presence of laboratory food source (bacterium *E. coli* OP50) even during toxin proteins exposure which avoided starvation-induced stress-responses in *C. elegans*. Hence, it appears that most, if not all the identified differentially regulated 601 proteins in our study are attributed to the PemK_Sa_ toxin interaction with the host system. This data infers *C. elegans* cells devote such a large portion of their proteins for defensive roles against toxins which also appear to cause membrane damage. The antioxidant enzymatic proteins were upregulated in treated samples might be to defend and counteract with ROS hydroxyl radicals (OH) and superoxide anions (O^2^-), and hydrogen peroxide (H_2_O_2_).

The highest confidence interaction map of downregulated proteins displayed the relationship between proteins responsible for generation and hydrolysis of ATP (ATP-2, VH-13), protein synthesis (eEF-2), carbohydrate metabolic process (MDH-2), fatty acid biosynthetic process (D1005.1), chaperonins (CCT-7), heat shock proteins (HSP), neuro transmission (GTA-1), and stress proteins (PDI). STRING analysis of upregulated proteins showed relationship between DAF-21, oxidative stress, protein folding, dephosphorylation, metabolic process, lipid transport activity, nutrient reservoir activity and oxidation-reduction process. Based on interactome analyses, the requirement of p38 and JNK MAPK pathways for PemK_Sa_ toxin defenses were examined. MAPK pathway is induced in mammalian and *C. elegans* cells by pore forming toxins PFTs (Stassen et al., 2003; Aguilar et al., 2009). RACK protein which is a multifaceted regulator and essential factor of *C. elegans* innate immunity against infection was downregulated in our study (Marudhupandiyan et al., 2017).

PemK_Sa_ toxin protein downregulates several molecular players related to lipid metabolism indicate alteration of *C. elegans* lipid metabolism. POD-2 encodes acetyl-CoA carboxylase, the rate limiting enzyme of *de novo* fatty acid biosynthesis (Shi et al., 2016). The ADS-1 (Alkyl-Dihydroxyacetone phosphate synthase) protein is an ortholog of human AGPS (Alkylglyceron phosphate synthase). The *ads-1* gene is encoding the protein required for ether lipid biosynthetic pathway initial steps (Shi et al., 2016). The *dhs-28* causes negative regulation of lipid storage (Joo et al., 2009). The *acbp-1* encodes acyl-CoA-binding protein required for lipid storage and transport (Elle et al., 2011). Several downregulated proteins responsible for lipid catabolic reactions, gluconeogenesis, carbohydrate degradation, and activation of pentose phosphate pathway predict degradation or peroxidation of lipids.

FT-IR data revealed the dramatically altered of *C. elegans* LPS constituents, the polysaccharide (1,250–1,150 cm^−1^), fatty acid (3,000–2,800 cm^−1^), and glycoprotein (1,200–900 cm^−1^) regions during the toxin exposure. Modification of *C. elegans* LPS and glycoproteins might be due to enhance the efficacy of the toxins or could be due to severity of toxin action on host. Glycosylation is a specific enzymatic process which involves many functional proteins resulting in a great diversity of carbohydrate-protein bonds and glycan structures. Various proteins responsible for the addition of sugars to the N- or the C-terminus of protein to form N- and O-glycans are found to be downregulated in this study (RIBO-1, OSTD-1, CRT-1, NMT-1, and LBP-1). RIBO-1 and OSTD-1 are an essential subunit of the N-oligosaccharyl transferase complex which catalyzes the transfer of a high mannose oligosaccharide from a lipid-linked oligosaccharide donor to an asparagine residue within an Asn-X-Ser/Thr consensus motif in nascent polypeptide chains.

Galectins are a group of lectins that bind β-galactoside-containing carbohydrates. The interaction of lectins with its target carbohydrate can be highly specific and allows this class of protein to participate in development, immunity, and cancer defense (Yang et al., 2008; Boscher et al., 2011). In addition, some of the *C. elegans* galectins have been confirmed to confer the diverse range of stress responses (Ideo et al., 2009). In this study, four galectins were regulated in which LEC-2, and LEC-4 were downregulated and LEC-1 and LEC-5 were upregulated at total protein, implying that galectin appears to be involved in the defense response of worm against toxin. Our studies are corroborated with the previous reports where it is stated that galectins (LEC-1, LEC-2, LEC-5, LEC-6, LEC-8, LEC-9, LEC-10, LEC-1081, and LEC-182) provide an immediate response during toxin exposure and oxidative stress (Griffitts et al., 2005; Ideo et al., 2009; Nemoto-Sasaki and Kasai, 2009; Takeuchi et al., 2013) Thus, our findings also supported the earlier studies on the regulatory roles of galectins of the host during the defense against toxins.

In this study, PemK_Sa_ intoxication has remarkably downregulated the expression level of HSP (HSP-3/6/12.2/60/70/110, ENPL-1, CCT-3/4/5/7/8, CYN-7, and GPD-3), whereas, HSP-4 and HSP-90 were highly abundant. *C. elegans* upon interaction with adverse environmental stimulator, bacterial infection and even physiological stressor, respond through rapid molecular changes for survival. HSPs are highly evolutionarily conserved molecular chaperones, which gives immediate responses during any stress, tissue damage, or bacterial infection (Frydman, 2001; Prithika et al., 2016; Wang et al., 2017). PemK_Sa_ intoxication has altered the expression level of HSPs and other proteins which play crucial role in pathological conditions by modulating the structurally denatured or misfolded proteins and prevent the proteins that retain its native conformation (Soti et al., 2005; Powers et al., 2010). Moreover, our *C. elegans* protein analysis during PemK_Sa_ exposure also identified stress-specific and immune response proteins (SODH-1, NHR-57, LYS-1, MOAC-1, and PYP-1), apoptosis-specific proteins (NDK-1, FEH-1, TGF-1, CDKR-3, DYN-1, and CCM-3) and another 35 proteins that are differentially regulated, all are responsible for cytoskeleton.

In conclusion, this study provided a preliminary view of molecular response of *C. elegans* toward a toxin at protein level, and provided insight into highly complex host-toxin interactions. Regulation of MAPK signal pathway, JNK, galectins, HSPs were observed for the first time to participate in the defense responses of worms against toxin proteins. This provided a novel insight into the mechanism of how eukaryotic system might respond to toxin. Our data indicate that PemK_Sa_ toxin causes paralysis, oxidative stress; protein misfolding and neuronal toxicity in the host system and triggers activation of cell death pathways.

## Author Contributions

DM designed and performed the experiments and analyzed the data. KB wrote the manuscript in consultation with DM.

### Conflict of Interest Statement

The authors declare that the research was conducted in the absence of any commercial or financial relationships that could be construed as a potential conflict of interest.

## Supplementary Material

Figure S1 **(A,B)** Transformation of pETDuet-PemK_Sa_ plasmid into *E. coli* BL21 (DE3) cells. Plasmids were inserted into *E. coli* BL21 (DE3) cells using calcium chloride mediated heat shock method. Transformed cells were selected in LB agar plates containing ampicillin (100 mg/L). plates-**A** contains, plates-**B** contains the DE3 transformed cells by pETDuet-PemK_Sa_ plasmid **(C)** Confirmation of pETDuet-PemK_Sa_ plasmid by restriction digestion. Lane-1 contains 1 kb ladder, lane-2 undigested pETDuet-PemK_Sa_ plasmid, and lane-3 pETDuet-PemK_Sa_ digested plasmid (by *EcoR1*, size: 5.5 kb). **(D)** Double digested plasmid by (*EcoR1* and *Sac1*), forms band at the position of 1,500 and 700 bp.

Figure S2 *C. elegans* total proteome (SDS-PAGE) after interaction with PemK_Sa_ toxin proteins. Lane (1 and 3) is N2 control sample exposed by *E. coli* OP50 and Lane (2 and 4) is N2 treated sample exposed by PemK_Sa_ toxin proteins.

Table S1 List of downregulated proteins present in *C. elegans* (control sample) identified using LC-MS/MS.

Table S2 List of upregulated proteins present in *C. elegans* (treated sample) identified using LC-MS/MS.
