# Supplementary figures and images for: Global Proteomic Response of Caenorhabditis elegans Against PemKSa Toxin
Source: Front Cell Infect Microbiol. 2019 May 31;9:172. doi: 10.3389/fcimb.2019.00172 (PMC6555269; doi:10.3389/fcimb.2019.00172)

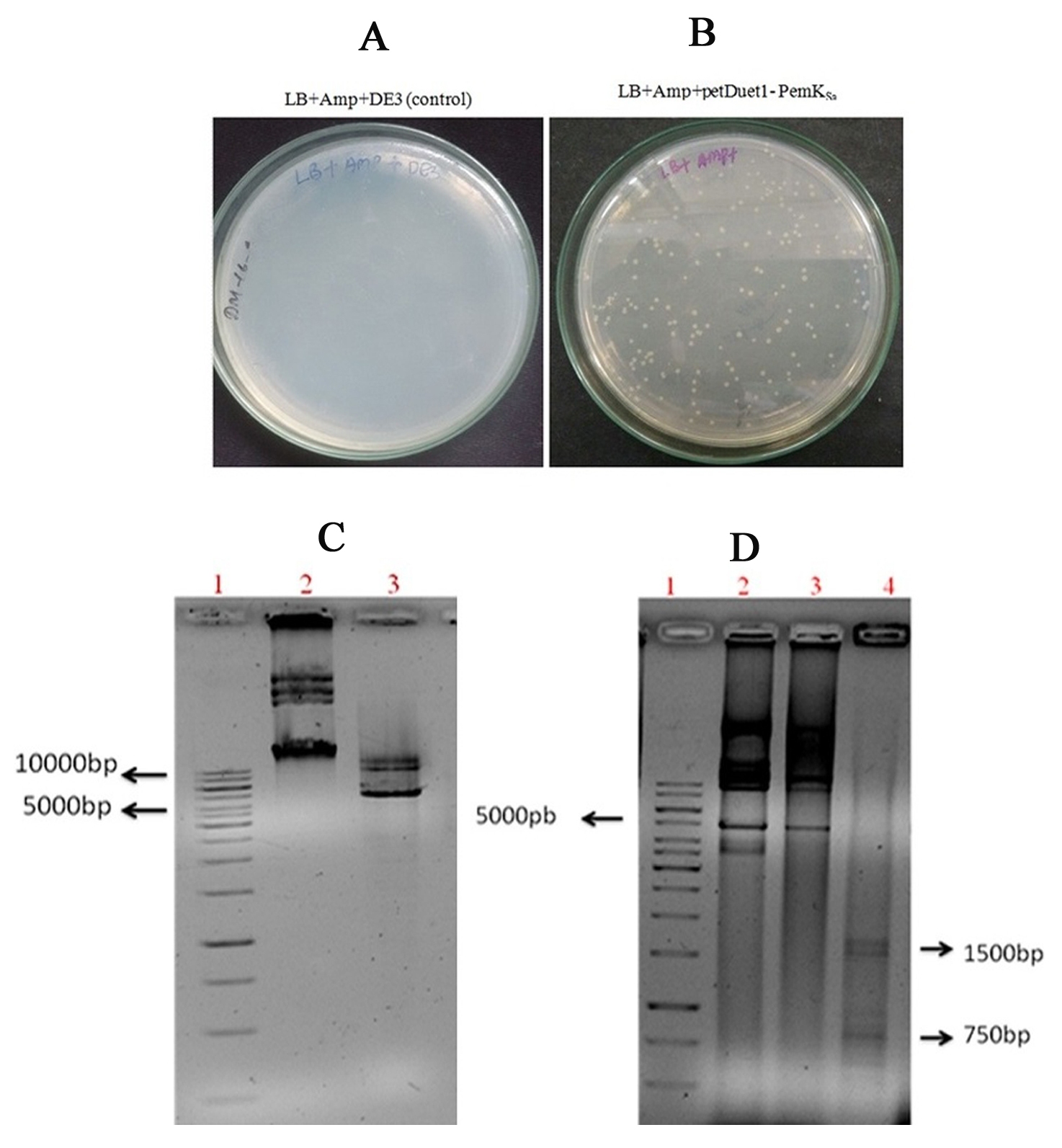

Supplement: Figure S1 — (A,B) Transformation of pETDuet-PemKSa plasmid into E. coli BL21 (DE3) cells. Plasmids were inserted into E. coli BL21 (DE3) cells using calcium chloride mediated heat shock method. Transformed cells were selected in LB agar plates containing ampicillin (100 mg/L). plates-A contains, plates-B contains the DE3 transformed cells by pETDuet-PemKSa plasmid (C) Confirmation of pETDuet-PemKSa plasmid by restriction digestion. Lane-1 contains 1 kb ladder, lane-2 undigested pETDuet-PemKSa plasmid, and lane-3 pETDuet-PemKSa digested plasmid (by EcoR1, size: 5.5 kb). (D) Double digested plasmid by (EcoR1 and Sac1), forms band at the position of 1,500 and 700 bp. [file Image_1.JPEG]

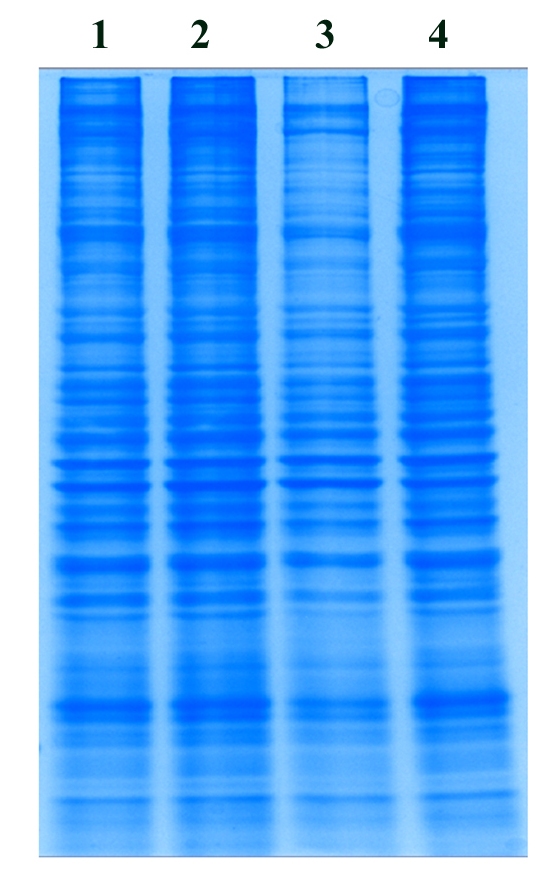

Supplement: Figure S2 — C. elegans total proteome (SDS-PAGE) after interaction with PemKSa toxin proteins. Lane (1 and 3) is N2 control sample exposed by E. coli OP50 and Lane (2 and 4) is N2 treated sample exposed by PemKSa toxin proteins. [file Image_2.JPEG]
