# Supplementary material for: Global Proteomic Response of Caenorhabditis elegans Against PemKSa Toxin
Source: Front Cell Infect Microbiol. 2019 May 31;9:172. doi: 10.3389/fcimb.2019.00172 (PMC6555269; doi:10.3389/fcimb.2019.00172)
Supplement: Table S1 — List of downregulated proteins present in C. elegans (control sample) identified using LC-MS/MS. [file Table_1.DOCX]

| Serial  **Table S1:** List of downregulated proteins present in *C. elegans* (control sample) identified using LC-MS/MS.  No | PROTEIN NAME | GENE | FOLD  CHANG | ANOVA |
| --- | --- | --- | --- | --- |
| 1 | Uncharacterized protein | ZK1073.1 | 5.2 | 0.0006 |
| 2 | Probable pyruvate dehydrogenase E1 component subunit | T05H10.6 | 4.8 | 0.0009 |
| 3 | UNC-44_ partial | unc-44 | 3.1 | 0.0007 |
| 4 | Uncharacterized protein | T19B10.2 | 6.6 | 0.0004 |
| 5 | Acyl-CoA-binding protein homolog 3 | acbp | 8.3 | 0.0004 |
| 6 | Transthyretin-like protein 15 | ttr-15 | 5.1 | 0.0005 |
| 7 | TransThyretin-Related family domain | ttr-41 | 4.6 | 0.0219 |
| 8 | Protein Up-regulated in Daf-2(gf) | pud-2.1 | 4.1 | 0.0173 |
| 9 | Uncharacterized helicase C28H8.3 | C28H8.3 | 5.7 | 0.0045 |
| 10 | Pyruvate dehydrogenase E1 component subunit beta | pdhb-1 | 3.6 | 0.0011 |
| 11 | Fatty acid-binding protein homolog 6 | lbp-6 | 3.6 | 0.0805 |
| 12 | Related to yeast Vacuolar Protein Sorting factor | vps-15 | 4.4 | 0.0001 |
| 13 | Protein phosphatase ppm-1 | ppm-1 | 2.9 | 0.0001 |
| 14 | Transforming acid coiled-coil-containing protein 1 | tac-1 | 4.0 | 0.0001 |
| 15 | Uncharacterized protein | Y6B3B.1 | 5.4 | 0.0001 |
| 16 | Galectin | lec-6 | 4.3 | 0.0001 |
| 17 | ATPase family AAA domain-containing protein 3 | atad-3 | 5.1 | 0.0001 |
| 18 | Uncharacterized protein | ZK1055.7 | 7.6 | 0.0001 |
| 19 | Dehydrogenases Short chain | dhs-20 | 3.5 | 0.0002 |
| 20 | Lipid Depleted | lpd-5 | 2.6 | 0.0002 |
| 21 | Importin Beta family | imb-3 | 3.0 | 0.0002 |
| 22 | Putative carbonic anhydrase 5 | cah-5 | 3.0 | 0.0002 |
| 23 | Transthyretin-like protein 46 | **ttr-46** | 7.4 | 0.0002 |
| 24 | Tubulin alpha-8 chain | tba-8 | 3.8 | 0.0003 |
| 25 | Uncharacterized protein | T20F5.4 | 2.5 | 0.0004 |
| 26 | Peptidylprolyl isomerase | CCD63089.1 | 4.2 | 0.0004 |
| 27 | Annexin | nex-3 | 3.8 | 0.0005 |
| 28 | TransThyretin-Related family domain | **ttr-48** | 3.3 | 0.0006 |
| 29 | Lipocalin-Related protein | lpr-3 | 3.1 | 0.0009 |
| 30 | Probable signal recognition particle subunit SRP68 | srpa-68 | 3.8 | 0.0007 |
| 31 | Formin Homology Domain | **fhod-1** | 2.7 | 0.0010 |
| 32 | V-type proton ATPase subunit a | vha-6 | 3.9 | 0.0010 |
| 33 | Regulator of Microtubule Dynamics | rmd-2 | 3.5 | 0.0013 |
| 34 | yeast SEC homolog | Sec-61 | 4.8 | 0.0013 |
| 35 | Rab-14 partial | **rab-14** | 2.8 | 0.0017 |
| 36 | CCDC (human Coiled Coil Domain Containing) homolog | **ccdc-47** | 2.9 | 0.0018 |
| 37 | cadmium-inducible lysosomal protein | cdr-4 | 3.3 | 0.0031 |
| 38 | Dehydrogenases_ Short chain | dhs-2 | 3.9 | 0.0037 |
| 39 | delta 12 fatty acid desaturase FAT-2 | fat-2 | 5.2 | 0.0037 |
| 40 | Uncharacterized protein | F15G9.2 | 4.1 | 0.0030 |
| 41 | Guanine nucleotide regulatory protein | rho-1 | 3.2 | 0.0040 |
| 42 | Uncharacterized protein | F21C3.6 | 8.4 | 0.0044 |
| 43 | Intramembrane protease 2 | imp-2 | 4.8 | 0.0056 |
| 44 | Peroxisomal Membrane Protein | pmp-5 | 5.0 | 0.0057 |
| 45 | Innexin-12 | inx-12 | 3.0 | 0.0158 |
| 46 | Uncharacterized protein | C55B7.10 | 3.8 | 0.0394 |
| 47 | Eukaryotic Initiation Factor | **eif-2** | 2.4 | 0.0411 |
| 48 | Inhibitor of Cell Death | icd-2 | 1.5 | 0.0060 |
| 49 | Elongation factor 1-alpha | eft-3 | 1.9 | 0.0828 |
| 50 | Uncharacterized protein | C47D2.1 | 3.2 | 0.0117 |
| 51 | NADH oxidoreductase complex I 23.8 kDa subunit | T20H4.5 | 21.8 | 0.0538 |
| 52 | kinesin like protein KLP-12 | klp-12 | 3.8 | 0.0123 |
| 53 | Fatty acid-binding protein homolog 5 | lbp-5 | 4.2 | 0.0130 |
| 54 | Mitogen activated protein kinase | Pmk-1 | 1.9 | 0.0028 |
| 55 | Elongation factor 1-alpha | eft-3 | 1.9 | 0.0082 |
| 56 | Elongation factor 2 | eef-2 | 1.9 | 0.0112 |
| 57 | Paramyosin | unc-15 | 1.8 | 0.0320 |
| 58 | Myosin-2 | myo-2 | 1.8 | 0.0129 |
| 59 | ATP synthase subunit beta_ mitochondrial | atp-2 | 1.9 | 0.0138 |
| 60 | Glutamate dehydrogenase | gdh-1 | 1.7 | 0.0307 |
| 61 | Probable arginine kinase F46H5.3 | F46H5.3 | 1.9 | 0.1717 |
| 62 | Actin-1 | act-1 | 1.7 | 0.0312 |
| 63 | Adenosylhomocysteinase | ahcy-1 | 1.8 | 0.0403 |
| 64 | Protein disulfide-isomerase 2 | pdi-2 | 1.6 | 0.0230 |
| 65 | Endoplasmin homolog | enpl-1 | 1.6 | 0.0178 |
| 66 | Probable clathrin heavy chain 1 | chc-1 | 1.6 | 0.0393 |
| 67 | Probable citrate synthase_ mitochondrial | cts-1 | 2.2 | 0.0495 |
| 68 | V-type proton ATPase catalytic subunit | vha-13 | 2.3 | 0.0074 |
| 69 | FiLamiN (Actin binding protein) homolog | fln-1 | 2.1 | 0.0267 |
| 70 | Glyceraldehyde-3-phosphate dehydrogenase 3 | gpd-3 | 1.5 | 0.0524 |
| 71 | La-related protein | larp-1 | 1.5 | 0.0203 |
| 72 | Probable malate dehydrogenase_ mitochondrial | mdh-2 | 1.5 | 0.0162 |
| 73 | Probable aconitate hydratase_ mitochondrial | aco-2 | 1.5 | 0.0485 |
| 74 | Heat shock 70 kDa protein C | hsp-3 | 1.7 | 0.0038 |
| 75 | Cytochrome b-c1 complex subunit 1_ mitochondrial | ucr-1 | 1.6 | 0.0278 |
| 76 | Calcium-transporting ATPase | sca-1 | 1.5 | 0.0480 |
| 77 | Guanine nucleotide-binding protein subunit beta-2-like 1 | rack-1 | 1.7 | 0.0471 |
| 78 | Phosphate carrier protein_ mitochondrial | F01G4.6 | 1.5 | 0.0237 |
| 79 | Intermediate filament protein ifb-1 | ifb-1 | 1.7 | 0.0098 |
| 80 | Isocitrate dehydrogenase [NADP] | idh-1 | 1.8 | 0.0196 |
| 81 | Calreticulin | crt-1 | 2.8 | 0.0017 |
| 82 | Probable voltage-dependent anion-selective channel | vdac-1 | 1.5 | 0.2445 |
| 83 | Histone H2A | his-3 | 2.2 | 0.0333 |
| 84 | Heat shock 70 kDa protein F_ mitochondrial | hsp-6 | 1.6 | 0.0843 |
| 85 | T-complex protein 1 subunit epsilon | cct-5 | 1.6 | 0.0772 |
| 86 | Probable elongation factor 1-gamma | eef-1G | 1.6 | 0.0429 |
| 87 | Uncharacterized protein | W08E12.7 | 1.6 | 0.0374 |
| 88 | Uncharacterized protein | Y69A2AR.18 | 1.6 | 0.0866 |
| 89 | Disorganized muscle protein 1 | dim-1 | 1.6 | 0.0585 |
| 90 | Probable V-type proton ATPase subunit B 1 | vha-12 | 1.6 | 0.0292 |
| 91 | Protein disulfide-isomerase | pdi-3 | 1.6 | 0.0156 |
| 92 | Uncharacterized protein | unc-52 | 1.6 | 0.0136 |
| 93 | Obg-like ATPase 1 | ola-1 | 1.6 | 0.0145 |
| 94 | Heat shock protein 110 | hsp-110 | 1.7 | 0.0097 |
| 95 | Protein disulfide-isomerase 1 | pdi-1 | 1.7 | 0.0572 |
| 96 | Tropomyosin isoforms a/b/d/f | lev-11 | 1.7 | 0.0663 |
| 97 | Ribosomal Protein_ Small subunit | rps-18 | 2.0 | 0.0016 |
| 98 | NucleOLar protein | nol-5 | 1.6 | 0.0373 |
| 99 | 2-oxoglutarate dehydrogenase_ mitochondrial | ogdh-1 | 1.7 | 0.0488 |
| 100 | Eukaryotic initiation factor 4A | inf-1 | 1.6 | 0.0120 |
| 101 | Probable methylmalonate-semialdehyde dehydrogenase | alh-8 | 1.8 | 0.0202 |
| 102 | Probable ATP-citrate synthase | acly-1 | 1.5 | 0.0541 |
| 103 | DAF-16/FOXO Controlled_ germline Tumor affecting | dct-16 | 1.8 | 0.0181 |
| 104 | Alcohol dehydrogenase 1 | sodh-1 | 1.5 | 0.0334 |
| 105 | Nucleosome Assembly Protein | nap-1 | 1.5 | 0.0465 |
| 106 | DeHydrogenases_ Short chain | dhs-28 | 1.6 | 0.0259 |
| 107 | Protein Up-regulated in Daf-2(Gf) | pud-2.1 | 1.8 | 0.0159 |
| 108 | ACTin | act-5 | 1.5 | 0.0370 |
| 109 | Heat shock 70 kDa protein D | hsp-4 | 1.5 | 0.0361 |
| 110 | T-complex protein 1 subunit delta | cct-4 | 1.6 | 0.0257 |
| 111 | Histone H4 | his-1 | 1.5 | 0.0341 |
| 112 | Propionyl Coenzyme A Carboxylase Beta subunit | pccb-1 | 1.6 | 0.0570 |
| 113 | Uncharacterized protein | Y37E3.8 | 1.8 | 0.0185 |
| 114 | Chaperonin Containing TCP-1 | cct-7 | 1.7 | 0.044 |
| 115 | Alpha-1_4 glucan phosphorylase | T22F3.3 | 1.8 | 0.0115 |
| 116 | Vacuolar H ATPase | vha-8 | 1.5 | 0.0592 |
| 117 | Propionyl-CoA carboxylase alpha chain | pcca-1 | 1.5 | 0.0281 |
| 118 | Probable 4-aminobutyrate aminotransferase | gta-1 | 1.6 | 0.0136 |
| 119 | ASpartyl Protease | asp-1 | 2.6 | 0.0046 |
| 120 | Very-long-chain 3-oxooacyl-coA reductase let-767 | let-767 | 1.7 | 0.0265 |
| 121 | Prolyl 4-hydroxylase subunit alpha- | dpy-18 | 1.5 | 0.0219 |
| 122 | ATP synthase subunit | atp-5 | 1.6 | 0.0173 |
| 123 | Uncharacterized protein | C18B2.5 | 2.2 | 0.0045 |
| 124 | Uncharacterized NOP5 family protein K07C5.4 | K07C5.4 | 1.6 | 0.0101 |
| 125 | Phosphoethanolamine MethylTransferase | pmt-2 | 1.8 | 0.0080 |
| 126 | Probable isocitrate dehydrogenase | idha-1 | 1.9 | 0.0057 |
| 127 | Ubiquinol-Cytochrome c oxidoReductase complex | ucr-2.1 | 1.5 | 0.0255 |
| 128 | Cystathionine Beta-Synthase | cbs-1 | 1.5 | 0.0443 |
| 129 | Aspartic protease 6 | asp-6 | 1.8 | 0.0061 |
| 130 | ATP synthase subunit | atp-4 | 2.1 | 0.0035 |
| 131 | Ribosomal Protein_ Small subunit | rps-22 | 1.6 | 0.0249 |
| 132 | Patterned Expression Site | pes-9 | 1.5 | 0.0189 |
| 133 | Adenine Nucleotide Translocator | ant-1.1 | 1.5 | 0.0270 |
| 134 | Cytochrome c 2.1 | cyc-2.1 | 2.2 | 0.0124 |
| 135 | IMportin Beta family | imb-3 | 1.5 | 0.0270 |
| 136 | Ribosomal Protein_ Large subunit | rpl-14 | 1.5 | 0.0152 |
| 137 | Bifunctional glyoxylate cycle protein | icl-1 | 1.5 | 0.0103 |
| 138 | Transcription factor BTF3 homolog | icd-1 | 1.8 | 0.0105 |
| 139 | Prion-like-(Q/N-rich)-domain-bearing protein | pqn-22 | 1.5 | 0.0198 |
| 140 | Four domain-type voltage-gated ion channel alpha-1 subunit | nca-2 | 1.8 | 0.0081 |
| 141 | 5C820 | pud-3 | 1.7 | 0.0229 |
| 142 | Nematode Polyprotein Allergen related | npa-1 | 1.6 | 0.0120 |
| 143 | Uncharacterized protein | T28D6.6 | 1.7 | 0.0589 |
| 144 | DAF-16/FOXO Controlled_ germline Tumor affecting | dct-18 | 1.5 | 0.0197 |
| 145 | Tubulin alpha-2 chain | tba-2 | 1.6 | 0.0491 |
| 146 | Fructose-bisphosphate aldolase 2 | aldo-2 | 1.6 | 0.0187 |
| 147 | Uncharacterized serine carboxypeptidase F41C3.5 | F41C3.5 | 1.6 | 0.0282 |
| 148 | Mitochondrial prohibitin complex protein 1 | phb-1 | 1.6 | 0.0109 |
| 149 | Uncharacterized protein | Y53G8AL.2 | 1.9 | 0.0051 |
| 150 | HAlF transporter (PGP related) | haf-4 | 1.5 | 0.0286 |
| 151 | Probable serine/threonine-protein phosphatase PP2A regulatory subunit | paa-1 | 1.7 | 0.0294 |
| 152 | Uncharacterized protein | C06A8.3 | 1.6 | 0.0013 |
| 153 | Uncharacterized protein | R05F9.6 | 1.7 | 0.0152 |
| 154 | Elongation factor 1-alpha | eft-3 | 1.5 | 0.0491 |
| 155 | Probable medium-chain specific acyl-CoA dehydrogenase | acdh-10 | 1.5 | 0.0312 |
| 156 | Probable electron transfer flavoprotein subunit alpha | F27D4.1 | 1.7 | 0.0905 |
| 157 | FumarylAcetoacetate Hydrolase | fah-1 | 1.8 | 0.0202 |
| 158 | Pyruvate kinase | pyk-1 | 1.6 | 0.0158 |
| 159 | Rab GDP dissociation inhibitor | gdi-1 | 1.8 | 0.0559 |
| 160 | Annexin | nex-1 | 1.6 | 0.0468 |
| 161 | Uncharacterized protein | pod-2 | 1.5 | 0.0429 |
| 162 | Uncharacterized protein | C44E4.4 | 1.6 | 0.0114 |
| 163 | Ribosomal Protein_ Small subunit | rps-10 | 1.7 | 0.0443 |
| 164 | Pyruvate dehydrogenase E1 component subunit beta_ mitochondrial | pdhb-1 | 1.5 | 0.0630 |
| 165 | Heat Shock Protein SV=1 | hsp-70 | 1.7 | 0.0762 |
| 166 | Proteasome Regulatory Particle_ ATPase-like 1 | rpt-5 | 1.5 | 0.0281 |
| 167 | Probable elongation factor 1-beta/1-delta 1 | eef-1B.1 | 1.5 | 0.0197 |
| 168 | Tubulin beta-2 chain | tbb-2 | 1.6 | 0.0390 |
| 169 | Transitional endoplasmic reticulum ATPase homolog 1 | cdc-48.1 | 1.5 | 0.0756 |
| 170 | Cytochrome c oxidase subunit 5A_ mitochondrial | cco-2 | 1.8 | 0.0480 |
| 171 | Intermediate filament protein ifa-1 | ifa-1 | 1.6 | 0.0118 |
| 172 | Elongation factor Tu homologue (Fragment) | **tufm-1** | 1.8 | 0.0579 |
| 173 | Proteasome Regulatory Particle_ Non-ATPase-like | rpn-1 | 1.6 | 0.0500 |
| 174 | Uncharacterized protein | Y71F9AL.9 | 1.8 | 0.0500 |
| 175 | Uncharacterized protein | spe-5 | 1.5 | 0.0521 |
| 176 | Uncharacterized protein | B0334.3 | 1.5 | 0.0495 |
| 177 | Aspartate aminotransferase | got-2.2 | 1.5 | 0.0598 |
| 178 | Probable ornithine aminotransferase_ mitochondrial | oatr-1 | 1.6 | 0.0149 |
| 179 | Probable ATP synthase subunit g 2_ mitochondrial | asg-2 | 1.8 | 0.0152 |
| 180 | Probable aspartate aminotransferase_ cytoplasmic | got-1.2 | 1.6 | 0.0113 |
| 181 | Uncharacterized protein | R07H5.8 | 1.9 | 0.0151 |
| 182 | NEPrilysin metallopeptidase family | nep-17 | 1.5 | 0.0582 |
| 183 | Cullin-associated NEDD8-dissociated protein 1 | cand-1 | 1.8 | 0.0240 |
| 184 | Protein Up-regulated in Daf-2(Gf) | pud-1.2 | 1.5 | 0.0175 |
| 185 | Intermediate Filament_ C | ifc-2 | 1.9 | 0.0017 |
| 186 | Rab-1 (Fragment) | rab-1 | 1.5 | 0.0450 |
| 187 | Histone H3 | his-40 | 1.8 | 0.0025 |
| 188 | Uncharacterized protein | C31C9.2 | 1.5 | 0.4017 |
| 189 | Dihydrolipoyl dehydrogenase_ mitochondrial | dld-1 | 1.5 | 0.5020 |
| 190 | Protein F37C4.5 | F37C4.5 | 1.8 | 0.0444 |
| 191 | Polyubiquitin-A | ubq-1 | 1.6 | 0.3621 |
| 192 | Tubulin beta-4 chain | tbb-4 | 1.6 | 0.0158 |
| 193 | T-complex protein 1 subunit alpha | cct-1 | 1.6 | 0.0238 |
| 194 | Transitional endoplasmic reticulum ATPase homolog 2 | cdc-48.2 | 1.6 | 0.0160 |
| 195 | CYtochrome P450 family | cyp-25a3 | 1.5 | 0.0533 |
| 196 | Probable elongation factor 1-beta/1-delta 2 | eef-1B.2 | 1.6 | 0.0234 |
| 197 | Adenine Nucleotide Translocator | ant-1.1 | 1.6 | 0.0233 |
| 198 | Excitatory amino acid transporter | glt-1 | 1.6 | 0.0112 |
| 199 | Sperm vesicle fusion protein fer-1 | fer-1 | 1.6 | 0.0064 |
| 200 | Acyl-CoA-binding protein homolog 3 | acbp-3 | 1.6 | 0.0193 |
| 201 | GaLECtin | lec-2 | 1.5 | 0.0493 |
| 202 | Eukaryotic translation initiation factor 5A-2 | iff-2 | 1.6 | 0.0114 |
| 203 | Uncharacterized protein | R04F11.2 | 1.5 | 0.0557 |
| 204 | Small Glutamine-rich Tetratrico repeat protein | sgt-1 | 1.6 | 0.0217 |
| 205 | Acetyl-CoA acetyltransferase homolog | kat-1 | 1.5 | 0.0539 |
| 206 | 26S proteasome regulatory subunit N6 | 01011 | 1.6 | 0.0435 |
| 207 | Ribosomal Protein_ Large subunit | rpl-34 | 1.8 | 0.0182 |
| 208 | Tyrosine-protein phosphatase | T13H5.1 | 1.5 | 0.0585 |
| 209 | Mitochondrial Carrier Homolog | mtch-1 | 1.8 | 0.0519 |
| 210 | Phosphoethanolamine Methyl Transferase | pmt-1 | 1.5 | 0.0391 |
| 211 | Uncharacterized protein | T23F2.2 | 1.7 | 0.0566 |
| 212 | Ribosomal Protein_ Large subunit | rpl-30 | 1.9 | 0.0051 |
| 213 | Acyl-CoA-binding protein homolog 1 | acbp-1 | 1.5 | 0.0538 |
| 214 | Intermediate filament protein ifa-2 | ifa-2 | 1.6 | 0.0331 |
| 215 | Uncharacterized protein | F44E7.4 | 1.5 | 0.0549 |
| 216 | Acid Alpha Glucosidase Relate | aagr-4 | 1.7 | 0.0202 |
| 217 | Cytochrome b-c1 complex subunit Rieske | isp-1 | 1.8 | 0.0158 |
| 218 | Phosphoenolypyruvate CarboxyKinase | pck-1 | 1.8 | 0.0435 |
| 219 | Tyrosine protein-kinase src-2 | src-2 | 1.6 | 0.0123 |
| 220 | Uncharacterized protein | Y45F10C.1 | 1.5 | 0.0582 |
| 221 | C. Elegans Y-box | cey-1 | 1.6 | 0.0323 |
| 222 | Uncharacterized protein | F56H1.3 | 2.1 | 0.0541 |
| 223 | Heat shock protein Hsp-16.48/Hsp-16.49 | hsp-16.48 | 1.8 | 0.0168 |
| 224 | Probable protein disulfide-isomerase A4 | C14B9.2 | 1.7 | 0.0125 |
| 225 | Heat shock protein Hsp-16.1/Hsp-16.11 | hsp-16.1 | 1.7 | 0.0582 |
| 226 | Putative cystathionine gamma-lyase 2 | cth-2 | 1.8 | 0.0581 |
| 227 | T-complex protein 1 subunit gamma | cct-3 | 1.8 | 0.0374 |
| 228 | Uncharacterized protein | ZK973.1 | 1.8 | 0.0345 |
| 229 | Zinc metalloproteinase nas-2 | nas-2 | 1.6 | 0.0002 |
| 230 | Uncharacterized protein | F49H12.5 | 1.5 | 0.0457 |
| 231 | Uncharacterized protein | Y57A10A.23 | 1.5 | 0.0248 |
| 232 | HnRNP A1 homolog | hrp-2 | 1.8 | 0.0124 |
| 233 | Coatomer subunit beta | copb-1 | 1.5 | 0.0399 |
| 234 | Yeast SEC homolog | sec-23 | 1.5 | 0.0498 |
| 235 | Translocon-Associated Protein | trap-4 | 1.5 | 0.0569 |
| 236 | Cytokinesis_ Apoptosis_ RNA-associated | car-1 | 1.8 | 0.0140 |
| 237 | KASH (Klarsicht/ANC-1/Syne Homology) Domain Protein | kdp-1 | 1.8 | 0.0103 |
| 238 | Triosephosphate isomerase | tpi-1 | 1.7 | 0.0415 |
| 239 | 6-phosphogluconate dehydrogenase_ decarboxylating | T25B9.9 | 1.6 | 0.0942 |
| 240 | 26S proteasome regulatory subunit 7 | rpt-1 | 1.6 | 0.0157 |
| 241 | Probable coatomer subunit beta' | copb-2 | 1.7 | 0.0139 |
| 242 | Probable 26S proteasome regulatory subunit rpn-6.1 | rpn-6.1 | 1.7 | 0.0495 |
| 243 | Regulator of Microtubule Dynamics | rmd-2 | 1.5 | 0.0386 |
| 244 | Uncharacterized protein | C51E3.9 | 1.6 | 0.0212 |
| 245 | NMDA class glutamate Receptor | nmr-2 | 1.6 | 0.0228 |
| 246 | Paralysed Arrest at Two-fold | pat-10 | 1.5 | 0.0450 |
| 247 | Cell death-related nuclease 2 | crn-2 | 1.7 | 0.0172 |
| 248 | Chaperonin Containing TCP-1 | cct-6 | 1.6 | 0.0701 |
| 249 | Nuclear Hormone Receptor family | nhr-246 | 1.5 | 0.0562 |
| 250 | Alanine--tRNA ligase_ cytoplasmic | aars-2 | 1.9 | 0.0003 |
| 251 | Fatty acid-binding protein homolog 6 | lbp-6 | 1.5 | 0.0347 |
| 252 | Nuclear hormone receptor family member nhr-57 | nhr-57 | 1.5 | 0.0180 |
| 253 | Yeast SEC homolog | sec-61 | 1.5 | 0.0520 |
| 254 | Proteasome subunit alpha type-1 | pas-6 | 1.6 | 0.0570 |
| 255 | Proline dehydrogenase 1 | B0513.5 | 1.5 | 0.0233 |
| 256 | MethylCrotonoyl-Coenzyme A Carboxylase (Alpha) | mccc-1 | 1.5 | 0.0518 |
| 257 | Proteasome Regulatory Particle_ Non-ATPase-like | rpn-8 | 1.7 | 0.0057 |
| 258 | Lysozyme-like protein | lys-1 | 1.9 | 0.0102 |
| 259 | Eukaryotic translation initiation factor 3 subunit D | eif-3.D | 1.6 | 0.0163 |
| 260 | V-type proton ATPase subunit D | vha-14 | 1.6 | 0.0540 |
| 261 | Probable glycylpeptide N-tetradecanoyltransferase | nmt-1 | 2.0 | 0.0137 |
| 262 | Putative aldehyde dehydrogenase family 7 member A1 homolog | alh-9 | 1.7 | 0.0249 |
| 263 | T-complex protein 1 subunit beta | cct-2 | 2.3 | 0.0026 |
| 264 | Peptidyl-prolyl cis-trans isomerase | cyn-7 | 1.5 | 0.0567 |
| 265 | Probable cytochrome c-type heme | cchl-1 | 1.8 | 0.0036 |
| 266 | Ribosomal Protein_ Small subunit | rps-29 | 1.6 | 0.0541 |
| 267 | Puromycin-sensitive aminopeptidase | pam-1 | 1.6 | 0.0179 |
| 268 | Uncharacterized protein | C47A10.12 | 1.9 | 0.0261 |
| 269 | TransALDolase-1 homolog | tald-1 | 1.6 | 0.0178 |
| 270 | Inosine-5'-monophosphate dehydrogenase | T22D1.3 | 1.7 | 0.0344 |
| 271 | Dolichyl-diphosphooligosaccharide--protein glycosyltransferase | ribo-1 | 1.8 | 0.0255 |
| 272 | Uncharacterized protein | Y39G8B.1 | 1.7 | 0.0554 |
| 273 | Hypoxanthine PhosphoRibosylTransferase homolog | hprt-1 | 1.5 | 0.0580 |
| 274 | TFG related | tfg-1 | 1.5 | 0.0567 |
| 275 | Histone H3.3 type 2 | his-72 | 1.6 | 0.0313 |
| 276 | Polyadenylate-binding protein | pab-1 | 1.8 | 0.0184 |
| 277 | Nicotinic receptor-associated protein 1 | nra-1 | 1.8 | 0.0392 |
| 278 | Eukaryotic translation initiation factor 3 subunit K | eif-3.K | 1.7 | 0.0184 |
| 279 | Ladder protein | CelDi5 | 1.8 | 0.0497 |
| 280 | Nascent polypeptide-associated complex subunit alpha | icd-2 | 1.7 | 0.0379 |
| 281 | Peptidylprolyl isomerase | fkb-5 | 1.7 | 0.0220 |
| 282 | Sodium/potassium-transporting ATPase subunit alpha | eat-6 | 3.6 | 0.0236 |
| 283 | Proteasomal ubiquitin receptor ADRM1 homolog | C56G2.7 | 1.5 | 0.0354 |
| 284 | ATP synthase subunit delta_ mitochondrial | F58F12.1 | 1.5 | 0.0542 |
| 285 | ADP-ribosylation factor 1-like 2 | arf-1.2 | 1.8 | 0.0092 |
| 286 | Uncharacterized protein | unc-44 | 1.7 | 0.0338 |
| 287 | Mitochondrial import receptor subunit TOM40 homolog | tomm-40 | 1.76 | 0.0107 |
| 288 | Galectin | lec-4 | 1.7 | 0.0165 |
| 289 | Cytochrome C | cyc-1 | 1.6 | 0.0024 |
| 290 | MAO-C-like dehydratase domain | maoc-1 | 1.7 | 0.0392 |
| 291 | CUTiclin-Like OS=Caenorhabditis elegans | cutl-5 | 1.6 | 0.0216 |
| 292 | Probable arginine--tRNA ligase_ cytoplasmic | rrt-1 | 1.7 | 0.0112 |
| 293 | Isocitrate dehydrogenase [NAD] subunit_ mitochondrial | idhg-1 | 1.8 | 0.0199 |
| 294 | Calsequestrin | csq-1 | 1.8 | 0.0124 |
| 295 | 14-3-3-like protein 2 | ftt-2 | 1.51 | 0.0562 |
| 296 | Probable cytochrome c oxidase subunit 6A_ mitochondrial | tag-174 | 1.5 | 0.0830 |
| 297 | NHP2-like protein 1 homolog | M28.5 | 1.8 | 0.0113 |
| 298 | Coronin-like protein cor-1 | cor-1 | 1.6 | 0.0337 |
| 299 | Proteasome Regulatory Particle_ Non-ATPase-like | rpn-9 | 1.9 | 0.0599 |
| 300 | Uncharacterized protein | T19B10.2 | 1.8 | 0.0578 |
| 302 | 4-hydroxyphenylpyruvate | hpd-1 | 2.5 | 0.0006 |
| 303 | Histone H2A.V | htz-1 | 1.5 | 0.0194 |
| 304 | Cysteine synthase 1 | cysl-1 | 1.5 | 0.0441 |
| 305 | Uncharacterized protein | NEDG_00927 | 1.7 | 0.0583 |
| 306 | Uncharacterized protein | NEDG_01035 | 4.6 | 0.0007 |
| 307 | Uncharacterized protein | NEDG_02216 | 2.1 | 0.0144 |
| 308 | Exonuclease 1 | NEDG_00233 | 3.0 | 0.0280 |
| 309 | Mannose-1-phosphate guanyltransferase beta | tag-335 | 2.2 | 0.0962 |
| 310 | LiPocalin-Related protein | lpr-7 | 2.2 | 0.0196 |
| 311 | ViGiLN homolog | vgln-1 | 1.5 | 0.0470 |
| 312 | Fructose-1_6-BiPhosphatase | fbp-1 | 2.7 | 0.0003 |
| 313 | NADPH-dependent diflavin oxidoreductase 1 1 | fre-1 | 1.6 | 0.0345 |
| 314 | Delta(9)-fatty-acid desaturase | fat-6 | 1.6 | 0.0235 |
| 315 | Cytochrome c oxidase subunit II | cox-2 | 2.3 | 0.0019 |
| 316 | Uncharacterized protein | Y37E3.17 | 2.2 | 0.0115 |
| 317 | Uncharacterized protein | F56B3.4 | 1.9 | 0.0211 |
| 318 | Uncharacterized protein | C03G5.14 | 2.0 | 0.0153 |
| 319 | Uncharacterized protein | F52D2.12 | 2.5 | 0.0342 |
| 320 | TransThyretin-Related family | ttr-9 | 1.6 | 0.0450 |
| 321 | Heat shock protein Hsp-16.41 | hsp-16.41 | 3.5 | 0.0072 |
| 322 | Casein kinase II subunit alpha | kin-3 | 1.7 | 0.0482 |
| 323 | Heat shock protein Hsp-12.2 | hsp-12.2 | 1.9 | 0.0057 |
| 324 | Putative serine protease K12H4.7 | K12H4.7 | 1.9 | 0.0274 |
| 325 | Probable 3-hydroxyacyl-CoA dehydrogenase B0272.3 | B0272.3 | 1.6 | 0.0157 |
| 326 | Tubulin alpha-8 chain | tba-8 | 1.5 | 0.0141 |
| 327 | Succinate--CoA ligase [ADP-forming] subunit | suca-1 | 1.6 | 0.0743 |
| 328 | Sodium/potassium-transporting ATPase subunit alpha | eat-6 | 1.6 | 0.0217 |
| 329 | Uncharacterized protein | C07D8.6 | 4.7 | 0.0097 |
| 330 | Dolichyl-diphosphooligosaccharide- protein glycosyltransferase | ostd-1 | 1.6 | 0.0432 |
| 331 | Probable phosphoglycerate kinase | pgk-1 | 2.0 | 0.0286 |
| 332 | Uncharacterized protein | F53F1.2 | 1.6 | 0.0349 |
| 333 | UDP-glucuronosyltransferase | ugt-23 | 1.7 | 0.0133 |
| 334 | PolyA Binding protein | pab-1 | 1.5 | 0.0466 |
| 335 | ALdehyde deHydrogenase | alh-12 | 1.7 | 0.0283 |
| 336 | Malate dehydrogenase | mdh-1 | 2.1 | 0.0286 |
| 337 | Piwi-like protein | wago-11 | 1.5 | 0.0370 |
| 338 | Thioredoxin | trx-4 | 1.5 | 0.0098 |
| 339 | Probable H/ACA ribonucleoprotein complex subunit | Y66H1A.4 | 1.6 | 0.0145 |
| 340 | F-box A protein | fbxa-23 | 1.5 | 0.0418 |
| 341 | Glycogen [starch] synthase | gsy-1 | 1.5 | 0.0352 |
| 342 | Uncharacterized protein | Y39G8B.2 | 1.7 | 0.0390 |
| 343 | Acyl carrier protein | Y56A3A.19 | 1.7 | 0.0155 |
| 344 | Profilin-1 | pfn-1 | 1.5 | 0.0227 |
| 345 | Putative phospholipase B-like 1 | Y37D8A.2 | 1.9 | 0.0432 |
| 346 | Putative H/ACA ribonucleoprotein complex subunit 2-like protein | Y48A6B.3 | 1.9 | 0.0492 |
| 347 | Homogentisate 1_2-dioxygenase | hgo-1 | 2.5 | 0.0165 |
| 348 | TRanslocon-Associated Protein | trap-1 | 3.6 | 0.0548 |
| 349 | Uncharacterized protein | C32D5.8 | 1.8 | 0.0003 |
| 350 | Uncharacterized protein | C16A3.5 | 1.7 | 0.0461 |
| 351 | SaPosin-like Protein family | spp-10 | 2.0 | 0.002 |
| 352 | Probable NADH dehydrogenase 1 alpha subcomplex subunit 5 | C33A12.1 | 1.7 | 0.0181 |
| 353 | Putative deoxyribose-phosphate aldolase | F09E5.3 | 1.8 | 0.0223 |
| 354 | Conserved Edge Expressed protein | cee-1 | 1.5 | 0.0224 |
| 355 | Uncharacterized protein | F35C11.6 | 1.7 | 0.0267 |
| 356 | Profilin-2 OS=Caenorhabditis elegans | pfn-2 | 1.5 | 0.0448 |
| 357 | Fatty Acid CoA Synthetase family | acs-11 | 2.5 | 0.0380 |
| 358 | Ras-Gtpase-activating protein SH3 (Three) domain-Binding Protein | gtbp-1 | 1.8 | 0.0333 |
| 359 | 2 (Zwei) IG domain protein | zig-12 | 1.6 | 0.0469 |
| 360 | Probable NADH dehydrogenase [ubiquinone] iron-sulfur protein 8 | T20H4.5 | 1.6 | 0.0043 |
| 361 | Proteasome subunit alpha type-5 | pas-5 | 1.5 | 0.0030 |
