# Supplementary material for: Global Proteomic Response of Caenorhabditis elegans Against PemKSa Toxin
Source: Front Cell Infect Microbiol. 2019 May 31;9:172. doi: 10.3389/fcimb.2019.00172 (PMC6555269; doi:10.3389/fcimb.2019.00172)
Supplement: Table S2 — List of upregulated proteins present in C. elegans (treated sample) identified using LC-MS/MS. [file Table_2.DOCX]

| Serial  **Table S2**: List of upregulated proteins present in *C. elegans* (treated sample) identified using LC/MS/MS.  No | PROTEIN NAME | GENE | FOLD  CHANG | ANOVA |
| --- | --- | --- | --- | --- |
| 1 | Phenylalanyl Amino-acyl tRNA Synthetase | fars-1 | 8.4 | 0.0030 |
| 2 | Uncharacterized protein | F13E6.1 | 5.0 | 0.0406 |
| 3 | Heavy chain, Unconventional Myosin | hum-2 | 4.0 | 0.0500 |
| 4 | Uncharacterized protein | B0511.11 | 109.9 | 0.0071 |
| 5 | 60S ribosomal protein L38 | **rpl-38** | 15.2 | 0.0335 |
| 6 | DNA helicase | **mcm-3** | 18.8 | 0.0421 |
| 7 | VIG (Drosophila Vasa Intronic Gene) ortholog | **vig-1** | 9.8 | 0.0001 |
| 8 | Glutathione S-Transferase | **gst-39** | 3.1 | 0.0001 |
| 9 | Acetyl-CoA Acyltransferase 2 homolog | **acaa-2** | 4.7 | 0.0027 |
| 10 | C. elegans Y-box | **cey-4** | 4.2 | 0.0113 |
| 11 | Ribosomal Protein Small subunit | **rps-6** | 21.9 | 0.0141 |
| 12 | Cysteinyl Amino-acyl tRNA Synthetase | **pdhb-1** | 6.1 | 0.0008 |
| 13 | Probable elongation factor 1-beta/1-delta 1 | **eef-1B.1** | 6.8 | 0.0163 |
| 14 | RIN (Ras/Rab Interactor) homolog | RIN-1 | 3.9 | 0.0001 |
| 15 | 40S ribosomal protein S6 | rps-6 | 11.8 | 0.0001 |
| 16 | Uncharacterized protein | D1054.10 | 4.1 | 0.0002 |
| 17 | Uncharacterized protein | Y82E9BR.14 | 6.1 | 0.0003 |
| 18 | Fructose-bisphosphate aldolase 2 | aldo-2 | 2.8 | 0.0003 |
| 19 | Dihydrolipoyl dehydrogenase_ mitochondrial | **dld-1** | 4.1 | 0.0005 |
| 20 | Uncharacterized protein | Y71F9AL.9 | 5.6 | 0.0006 |
| 21 | Permeable eggshell | **perm-2** | 5.0 | 0.0007 |
| 22 | Uncharacterized protein | F49D11.10 | 6.6 | 0.0008 |
| 23 | unnamed protein product | CBA11166.1 | 4.1 | 0.0009 |
| 24 | Broad/complex/Tramtrack/Bric a brac domain protein | **btb-17** | 2.7 | 0.0010 |
| 25 | rabGDI | **gdi-1** | 8.8 | 0.0011 |
| 26 | Probable ribosome biogenesis protein RLP24 | **rpl-24.2** | 3.5 | 0.0024 |
| 27 | Calcium/calmodulin-dependent protein kinase type 1 | **cmk-1** | 3.8 | 0.0025 |
| 28 | Inactive tyrosine-protein kinase kin-32 | **kin-32** | 6.9 | 0.0027 |
| 29 | Uncharacterized protein | R05H5.3 | 3.0 | 0.0049 |
| 30 | Collagen | **col-71** | 5.8 | 0.0163 |
| 31 | Ribosomal Protein Small subunit | **rps-24** | 3.5 | 0.0170 |
| 32 | heat shock protein 70A | hsp-70 | 3.4 | 0.0420 |
| 33 | 40S ribosomal protein S23 | **rps-23** | 2.8 | 0.0390 |
| 34 | Uncharacterized protein | M01H9.3 | 1.5 | 0.0960 |
| 35 | Alpha-1_4 glucan phosphorylase | T22F3.3 | 1.8 | 0.0115 |
| 36 | Vacuolar H ATPase | vha-8 | 1.7 | 0.0030 |
| 37 | 60S ribosomal protein L9 | rpl-9 | 1.8 | 0.0411 |
| 38 | 60S ribosomal protein L7a | rpl-7 | 1.8 | 0.0287 |
| 39 | Probable inorganic pyrophosphatase 1 | pyp-1 | 1.5 | 0.0560 |
| 40 | Translationally-controlled tumor protein homolog | tct-1 | 1.9 | 0.0166 |
| 41 | VIG (Drosophila Vasa Intronic Gene) ortholog | vig-1 | 1.9 | 0.0298 |
| 42 | Pyruvate carboxylase 1 | pyc-1 | 1.8 | 0.0511 |
| 43 | Xaa-Pro aminopeptidase app-1 | app-1 | 1.7 | 0.0610 |
| 44 | 14-3-3-like protein 1 | par-5 | 1.8 | 0.0225 |
| 45 | 40S ribosomal protein S8 | rps-8 | 1.5 | 0.0461 |
| 46 | Probable protein disulfide-isomerase A6 | tag-320 | 1.8 | 0.0514 |
| 47 | ATP Synthase B homolog | asb-2 | 1.7 | 0.0561 |
| 48 | Glutamyl(E) Amino-acyl tRNA Synthetase | ears-1 | 1.5 | 0.0400 |
| 49 | Sodium/potassium-transporting ATPase subunit beta-1 | nkb-1 | 1.9 | 0.0286 |
| 50 | UBA (Human ubiquitin) related | uba-1 | 1.8 | 0.0058 |
| 51 | 60S acidic ribosomal protein P2 | C37A2.7 | 1.8 | 0.0530 |
| 52 | Uncharacterized protein | R02D3.1 | 1.8 | 0.0528 |
| 53 | Vitellogenin-2 | vit-2 | 1.6 | 0.0529 |
| 54 | 40S ribosomal protein S5 | rps-5 | 1.5 | 0.0113 |
| 55 | Vitellogenin-1 | vit-1 | 1.9 | 0.0083 |
| 56 | 60S ribosomal protein L12 | rpl-12 | 1.9 | 0.0133 |
| 57 | Unconventional myosin heavy chain 6 | hum-6 | 1.9 | 0.0832 |
| 58 | DEAD boX helicase homolog | ddx-17 | 1.7 | 0.0389 |
| 59 | Ribosomal Protein_ Small subunit | rps-11 | 1.6 | 0.0815 |
| 60 | rRNA 2'-O-methyltransferase fibrillarin | fib-1 | 1.8 | 0.0472 |
| 61 | Probable 26S proteasome regulatory subunit 10B | rpt-4 | 1.7 | 0.0325 |
| 62 | DihydroLipoamide S-SuccinylTransferase | dlst-1 | 1.6 | 0.0421 |
| 63 | Eukaryotic Release FActor homolog | erfa-3 | 1.9 | 0.0396 |
| 64 | Eukaryotic translation initiation factor 3 subunit A | egl-45 | 1.8 | 0.0466 |
| 65 | Uncharacterized protein | F36A2.7 | 1.5 | 0.0324 |
| 66 | Ribosomal Protein Small subunit | rps-20 | 1.7 | 0.0481 |
| 67 | 60S ribosomal protein L3 | rpl-3 | 1.8 | 0.0470 |
| 68 | Transcription initiation factor TFIID subunit 2 | taf-2 | 1.8 | 0.0238 |
| 69 | NADH Ubiquinone Oxidoreductase | nuo-5 | 1.8 | 0.0559 |
| 70 | Eukaryotic translation initiation factor 3 subunit F | eif-3.F | 1.6 | 0.0140 |
| 71 | Troponin T | mup-2 | 1.6 | 0.0593 |
| 72 | 2-oxoisovalerate dehydrogenase subunit alpha | bckd-1A | 1.8 | 0.0057 |
| 73 | Vitellogenin-5 | vit-5 | 1.8 | 0.0194 |
| 74 | Myosin regulatory light chain 1 | mlc-1 | 1.9 | 0.0437 |
| 75 | Probable pyruvate dehydrogenase E1 component subunit | T05H10.6 | 1.8 | 0.0038 |
| 76 | Fructose-bisphosphate aldolase 1 | aldo-1 | 1.9 | 0.0517 |
| 77 | TropoNin T | tnt-2 | 1.8 | 0.0103 |
| 78 | Probable sodium/potassium-transporting ATPase subunit | nkb-3 | 1.7 | 0.0217 |
| 79 | Leucine--tRNA ligase | lrs-1 | 1.8 | 0.0506 |
| 80 | ViGiLN homolog | vgln-1 | 1.6 | 0.0225 |
| 81 | ALdehyde deHydrogenase | alh-1 | 1.8 | 0.0524 |
| 82 | MYOsin heavy chain structural genes | myo-5 | 1.9 | 0.0106 |
| 83 | EXPOrtin (Nuclear export receptor) | xpo-1 | 1.8 | 0.0236 |
| 84 | Probable V-type proton ATPase subunit F | vha-9 | 1.7 | 0.0318 |
| 85 | Uncharacterized protein | VF13D12L.3 | 1.8 | 0.0134 |
| 86 | Galectin | lec-5 | 1.5 | 0.0433 |
| 87 | Delta(12) fatty acid desaturase fat-2 | fat-2 | 1.8 | 0.0438 |
| 88 | Plant Late Embryo Abundant (LEA) related | lea-1 | 1.9 | 0.0345 |
| 89 | Uncharacterized protein | T23E7.2 | 1.8 | 0.0400 |
| 90 | Uncharacterized protein | ZK488.5 | 1.6 | 0.0350 |
| 91 | ATP synthase subunit | atp-3 | 1.6 | 0.0534 |
| 92 | Uncharacterized protein | H24G06.1 | 1.5 | 0.0461 |
| 93 | NADH dehydrogenase [ubiquinone] 1 alpha subcomplex | Y54F10AM.5 | 1.5 | 0.0275 |
| 94 | Uncharacterized protein | Y54E10A.6 | 1.6 | 0.0488 |
| 95 | Coatomer subunit alpha | copa-1 | 1.5 | 0.0724 |
| 96 | Vitellogenin-3 | vit-3 | 1. | 0.0399 |
| 97 | Uncharacterized protein | Y22D7AL.10 | 1.5 | 0.0291 |
| 98 | Uncharacterized protein | R09H10.5 | 1.5 | 0.0020 |
| 99 | Probable isocitrate dehydrogenase [NAD] | idhb-1 | 2.7 | 0.0030 |
| 100 | Myosin-4 | unc-54 | 1.9 | 0.0031 |
| 101 | Glutathione reductase_ mitochondrial | gsr-1 | 1.5 | 0.0390 |
| 102 | Probable NADH dehydrogenase | nduf-7 | 1.5 | 0.0212 |
| 103 | V-type proton ATPase catalytic subunit A | vha-13 | 1.5 | 0.0570 |
| 104 | Uncharacterized protein | daf-3 | 1.6 | 0.0161 |
| 105 | Uncharacterized protein | 01589 | 1.6 | 0.0135 |
| 106 | CCR4-NOT transcription complex subunit 1 | 00266 | 1.8 | 0.0029 |
| 107 | Uncharacterized protein | H14E04.2 | 1.6 | 0.0567 |
| 108 | 60S ribosomal protein L24 | rpl-24.1 | 1.6 | 0.0242 |
| 109 | Fatty Acid CoA Synthetase family | acs-10 | 1.5 | 0.0519 |
| 110 | Uncharacterized protein | C29F7.2 | 1.6 | 0.0348 |
| 111 | Uncharacterized protein | F42G8.10 | 1.5 | 0.0250 |
| 112 | C-type LECtin | clec-63 | 1.5 | 0.0370 |
| 113 | Dynamin | dyn-1 | 1.6 | 0.0277 |
| 114 | Uncharacterized protein | C50F2.2 | 1.8 | 0.0207 |
| 115 | Phenylalanyl Amino-acyl tRNA Synthetase | fars-1 | 1.8 | 0.0581 |
| 116 | T-complex protein 1 subunit gamma | cct-3 | 1.8 | 0.0345 |
| 117 | Uncharacterized protein | ZK973.1 | 1.8 | 0.0437 |
| 118 | SURF1-like protein | sft-1 | 2.3 | 0.0005 |
| 119 | Uncharacterized protein | C38D9.8 | 1.5 | 0.0483 |
| 120 | Myosin-2 essential light chain | mlc-5 | 1.5 | 0.0207 |
| 121 | Uncharacterized protein | F41H10.12 | 1.5 | 0.0422 |
| 122 | Probable glutathione S-transferase 5 | gst-5 | 1.8 | 0.0100 |
| 123 | Probable glutathione S-transferase gst-36 | gst-36 | 1.5 | 0.0536 |
| 124 | RutC family protein C23G10.2 | C23G10.2 | 1.7 | 0.0028 |
| 125 | Uncharacterized protein | B0491.5 | 1.6 | 0.0196 |
| 126 | Dihydropyrimidine dehydrogenase [NADP(+)] | dpyd-1 | 2.6 | 0.0003 |
| 127 | Protein FAM50 homolog | C47E8.4 | 1.6 | 0.0292 |
| 128 | ABC transporter_ class F | abcf-3 | 1.5 | 0.0113 |
| 129 | Putative ATP synthase subunit | R53.4 | 1.7 | 0.0141 |
| 130 | CaLponIn-liKe proteins | clik-1 | 1.8 | 0.0386 |
| 131 | YTochrome B | cytb-5.1 | 1.5 | 0.0250 |
| 132 | Unc-87 protein | unc-87 | 1.6 | 0.0276 |
| 133 | Uncharacterized protein | 00918 | 1.7 | 0.0208 |
| 134 | Kinesin family member 2/24 | 00250 | 1.7 | 0.0533 |
| 135 | Uncharacterized protein | C41G7.9 | 1.5 | 0.0513 |
| 136 | Ribosomal Protein_ Large subunit | rpl-32 | 2.1 | 0.0082 |
| 137 | UDP-glucuronosyltransferase | ugt-16 | 1.7 | 0.0141 |
| 138 | CaLponIn-liKe proteins | clik-1 | 1.5 | 0.1774 |
| 139 | Carboxypeptidase | Y16B4A.2 | 1.6 | 0.0403 |
| 140 | Serpentine Receptor_ class SX | srsx-18 | 1.6 | 0.0960 |
| 141 | Uncharacterized protein | F22E5.13 | 1.6 | 0.0770 |
| 142 | Proline dehydrogenase 1_ mitochondrial | B0513.5 | 1.6 | 0.0574 |
| 143 | Ref/ALY RNA export adaptor family | aly-2 | 1.5 | 0.0600 |
| 144 | Mitochondrial Ribosomal Protein_ Large | mrpl-28 | 1.5 | 0.0402 |
| 145 | Myosin regulatory light chain 2 | mlc-2 | 1.5 | 0.0486 |
| 146 | cAMP-dependent protein kinase regulatory subunit | kin-2 | 1.8 | 0.0005 |
| 147 | Ubiquitin-like protein 1-40S ribosomal protein S27a | ubl-1 | 1.6 | 0.0132 |
| 148 | Threonine tRNA ligase cytoplasmic | trs-1 | 1.5 | 0.0456 |
| 149 | N-acetylgalactosaminyl transferase 7 | GalNAc-T2 | 1.9 | 0.0108 |
| 150 | T-complex protein 1 subunit theta | cct-8 | 1.5 | 0.0416 |
| 151 | Uncharacterized protein | Y55F3AM.13 | 2.1 | 0.0195 |
| 152 | Kinesin like protein | klp-17 | 1.6 | 0.0305 |
| 153 | NADH dehydrogenase [ubiquinone] 1 alpha subcomplex | NDUFA13 | 2.2 | 0.0248 |
| 154 | ALdehyde deHydrogenase | alh-2 | 1.9 | 0.0318 |
| 155 | Dolichol Phosphate Mannosyl transferase | dpm-1 | 1.6 | 0.0002 |
| 156 | Polycystin-2 | pkd-2 | 1.7 | 0.0002 |
| 157 | V-type proton ATPase subunit C | vha-11 | 1.5 | 0.0480 |
| 158 | Mitochondrial Ribosomal Protein Large | mrpl-9 | 1.6 | 0.0196 |
| 159 | Proteasome subunit alpha type-3 | pas-7 | 1.7 | 0.0057 |
| 160 | Uncharacterized protein C53C9.2 | C53C9.2 | 1.5 | 0.0588 |
| 161 | Dosage compensation protein dpy-30 | dpy-30 | 1.6 | 0.0111 |
| 162 | Homeobox protein ceh-40 | ceh-40 | 1.5 | 0.0328 |
| 163 | Acetoacetyl-CoA synthetase | sur-5 | 1.5 | 0.0367 |
| 164 | Probable peroxiredoxin prdx-3 | prdx-3 | 2.0 | 0.0061 |
| 165 | Uncharacterized protein | T08H10.1 | 2.3 | 0.0286 |
| 166 | Kinetochore protein Nuf2 1 | 01080 | 1.5 | 0.0134 |
| 167 | Uncharacterized protein | 01141 | 3.1 | 0.0058 |
| 168 | Uncharacterized protein | 01788 | 1.7 | 0.0485 |
| 169 | Uncharacterized protein | Y105E8A.20 | 1.5 | 0.0472 |
| 170 | Translationally controlled tumor protein | tctp | 1.7 | 0.0365 |
| 171 | Uncharacterized protein | C02D5.4 | 1.5 | 0.0292 |
| 172 | Eukaryotic translation initiation factor 5A | iff-1 | 3.6 | 0.0063 |
| 173 | AANAT (Arylalkylamine N-AcetylTransferase) homolog | anat-1 | 1.8 | 0.0172 |
| 174 | Cytochrome P450 family | cyp-34a9 | 1.5 | 0.0239 |
| 175 | Galectin | lec-3 | 1.5 | 0.0484 |
| 176 | Uncharacterized protein | C32F10.8 | 1.6 | 0.0482 |
| 177 | Uncharacterized protein | F45H10.3 | 1.8 | 0.0011 |
| 178 | Uncharacterized protein | T06D4.2 | 1.6 | 0.0281 |
| 179 | Uncharacterized protein | C49F5.7 | 1.8 | 0.0051 |
| 180 | Serine/threonine-protein phosphatase 2A subunit | pptr-1 | 1.7 | 0.0158 |
| 181 | Serpentine Receptor class H | srh-59 | 1.6 | 0.0169 |
| 182 | Uncharacterized protein | F59B1.2 | 1.5 | 0.0309 |
| 183 | Glyceraldehyde-3-phosphate dehydrogenase | gpd-1 | 1.5 | 0.0577 |
| 184 | Actin-2 | act-2 | 1.8 | 0.0364 |
| 185 | Vitellogenin-4 | vit-4 | 1.9 | 0.0002 |
| 186 | Fatty-acid and retinol-binding protein 2 | far-2 | 2.4 | 0.0019 |
| 187 | Serine/threonine-protein phosphatase PP1-beta | gsp-2 | 1.5 | 0.0401 |
| 188 | Probable V-type proton ATPase subunit G | vha-10 | 1.5 | 0.0530 |
| 189 | Uncharacterized protein | F59A3.2 | 1.5 | 0.0425 |
| 190 | GRound-Like (Grd related) | grl-27 | 1.5 | 0.0321 |
| 191 | Ferritin | ftn-2 | 1.5 | 0.0218 |
| 192 | Vacuolar H ATPase | vha-19 | 1.5 | 0.0258 |
| 193 | Malate dehydrogenase | mdh-1 | 1.7 | 0.0543 |
| 194 | Uncharacterized protein | D1014.6 | 1.5 | 0.0105 |
| 195 | Putative serine/threonine-protein kinase R03D7.5 | R03D7.5 | 1.9 | 0.0597 |
| 196 | Uncharacterized protein T28D9.1 | T28D9.1 | 3.5 | 0.0057 |
| 197 | Programmed cell death protein 10 homolog | ccm-3 | 2.0 | 0.0027 |
| 198 | Related to yeast Vacuolar Protein Sorting factor | vps-32.1 | 2.9 | 0.0161 |
| 199 | Uncharacterized protein | E02C12.11 | 1.9 | 0.058 |
| 200 | F-box A protein | fbxa-53 | 1.6 | 0.0191 |
| 201 | Adenylate kinase isoenzyme 1 | F38B2.4 | 1.6 | 0.0214 |
| 202 | Uncharacterized protein | F49C12.4 | 2.4 | 0.0058 |
| 203 | Probable NADH dehydrogenase [ubiquinone] flavoprotein | F53F4.10 | 2.3 | 0.0137 |
| 204 | Uncharacterized protein | T02G5.7 | 1.5 | 0.0251 |
| 205 | Ubiquinol-Cytochrome c oxidoReductase complex | ucr-2.2 | 1.6 | 0.0499 |
| 206 | Uncharacterized protein | rad-23 | 3.3 | 0.0027 |
| 207 | Putative aminopeptidase W07G4.4 | lap-2 | 3.5 | 0.0057 |
| 208 | Myosin-4 | unc-54 | 1.7 | 0.0582 |
| 209 | Vitellogenin-6 | vit-6 | 1.6 | 0.0243 |
| 210 | Heat shock 70 kDa protein A | hsp-1 | 1.9 | 0.0098 |
| 211 | Myosin-1 | myo-1 | 1.6 | 0.0113 |
| 212 | ATP synthase subunit alpha | H28O16.1 | 1.7 | 0.0335 |
| 213 | Heat shock protein 90 | daf-21 | 1.5 | 0.0423 |
| 214 | Myosin-3 | myo-3 | 1.6 | 0.0298 |
| 215 | 60S ribosomal protein L7 | rpl-7 | 1.6 | 0.0242 |
| 216 | 60S ribosomal protein L5 | rpl-5 | 1.5 | 0.0274 |
| 217 | Tudor Staphylococcal Nuclease homolog | tsn-1 | 1.6 | 0.0128 |
| 218 | Myosin essential light chain | mlc-3 | 1.5 | 0.9615 |
| 219 | Chaperonin homolog Hsp-60 mitochondrial | hsp-60 | 1.9 | 0.0139 |
| 220 | 40S ribosomal protein S3a | rps-1 | 1.6 | 0.0692 |
| 221 | 40S ribosomal protein S2 | rps-2 | 1.5 | 0.0318 |
| 222 | Probable cytoplasmic aconitate hydratase | aco-1 | 1.5 | 0.0581 |
| 223 | 60S ribosomal protein L19 | rpl-19 | 1.5 | 0.0115 |
| 224 | PeRoxireDoXin | prdx-2 | 1.7 | 0.0205 |
| 225 | Intermediate Filament B | ifb-2 | 1.9 | 0.0478 |
| 226 | 60S ribosomal protein L27 | rpl-27 | 1.8 | 0.0015 |
| 227 | 60S ribosomal protein L13a | rpl-16 | 1.8 | 0.0096 |
| 228 | 32 kDa beta-galactoside-binding lectin | lec-1 | 1.6 | 0.0288 |
| 229 | Enoyl-CoA Hydratase | ech-1.2 | 1.8 | 0.0369 |
| 230 | Valine--tRNA ligase | vrs-2 | 1.9 | 0.0640 |
| 231 | ACEtyl-CoA Regulator | acer-1 | 1.8 | 0.0510 |
| 232 | 40S ribosomal protein S9 | rps-9 | 1.7 | 0.0126 |
| 233 | Isoleucine-tRNA ligase cytoplasmic | irs-1 | 1.6 | 0.0460 |
| 234 | Protein unc-87 | unc-87 | 1.5 | 0.0207 |
| 235 | 40S ribosomal protein SA | rps-0 | 1.8 | 0.0291 |
| 236 | 60S ribosomal protein L35a | rpl-33 | 1.5 | 0.0050 |
| 237 | 40S ribosomal protein S12 | rps-12 | 1.84 | 0.0216 |
| 238 | Probable S-adenosylmethionine synthase 1 | sams-1 | 1.6 | 0.0044 |
| 239 | 40S ribosomal protein S14 | rps-14 | 1.5 | 0.0048 |
| 240 | 60S ribosomal protein L8 | rpl-8 | 1.5 | 0.0308 |
